# Regioselective synthesis of 7,8-dihydroimidazo[5,1-*c*][1,2,4]triazine-3,6(2*H*,4*H*)-dione derivatives: A new drug-like heterocyclic scaffold

**DOI:** 10.3762/bjoc.8.181

**Published:** 2012-09-20

**Authors:** Nikolay T Tzvetkov, Harald Euler, Christa E Müller

**Affiliations:** 1 PharmaCenter Bonn, Pharmaceutical Sciences Bonn (PSB), University of Bonn, Pharmaceutical Institute, An der Immenburg 4, 53121 Bonn, Germany; 2 University of Bonn, Steinmann-Institute of Mineralogy, Poppelsdorfer Schloss, 53115 Bonn, Germany

**Keywords:** hydantoins, hydrazides, imidazotriazines, N-alkylation, regio- and chemoselective reaction, thionation, X-ray structure

## Abstract

Dihydroimidazo[5,1-*c*][1,2,4]triazine-3,6(2*H*,4*H*)-dione derivatives were prepared by successive N3- and N1-alkylation of hydantoins, followed by regioselective thionation and subsequent cyclization under mild conditions. In a final alkylation step a further substituent may be introduced. The synthetic strategy allows broad structural variation of this new drug-like heterobicyclic scaffold. In addition to extensive NMR and MS analyses, the structure of one derivative was confirmed by X-ray crystallography.

## Introduction

**Figure 1:**
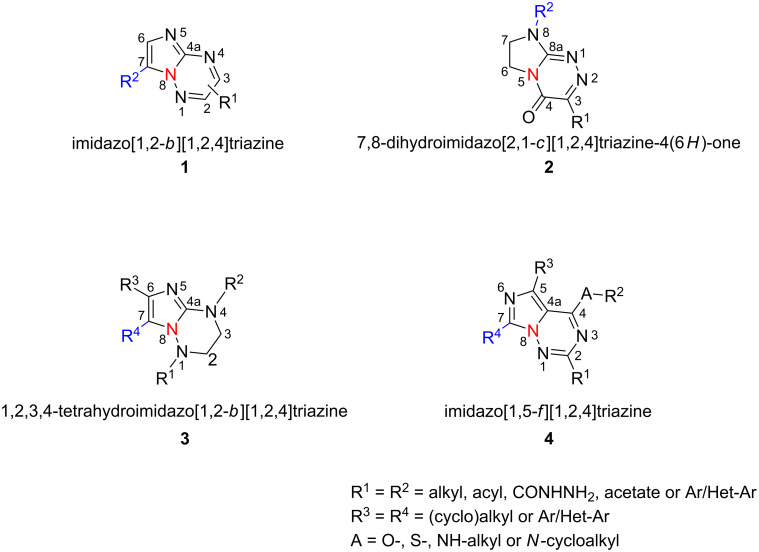
Biologically active imidazo[1,2,4]triazine scaffolds **1**–**4**.

Imidazotriazines represent an important class of condensed heterobicycles that display a variety of significant biological activities, including anticancer [1], antimicrobial [2], anti-inflammatory [3] and neuroprotective [4] properties. The impressive array of biological effects of these compounds is associated with the 1,2,4-triazine ring as the core structural moiety, which also occurs in a number of natural products [5]. In addition, the 1,2,4-triazine scaffold has found application in pharmaceuticals and agrochemicals [6]. For example, some 7-phenylimidazo-[1,2.*b*][1,2,4]triazine derivatives of the general structure **1** (Figure 1) have been developed as selective ligands for γ-aminobutyric acid type A (GABA_A_) receptors and are therefore of benefit in the treatment and prevention of adverse conditions of the central nervous system (CNS), including anxiety, convulsions and cognitive disorders [7]. Novel fused 1,2,4-triazine-4(6*H*)-ones **2** showed selective cytotoxicity at micromolar concentrations against a wide range of cancer cells [1,8], while other derivatives of **2** exhibited analgesic [9], antibacterial, and antiviral activities [2]. Compounds with the 1,2,3,4-tetrahydroimidazo[1,2-*b*][1,2,4]triazine framework **3** were reported to be potentially useful as interleukin-1 (IL-1) and tumour necrosis factor (TNF) inhibitors for prophylactic and therapeutic treatment of chronic inflammatory diseases in which these cytokines are involved [3]. Recently, specifically substituted imidazo[1,5-*f*][1,2,4]triazines (**4**) have been developed as polo-like kinase (PLK) inhibitors with potential as anticancer therapeutics [10]. The 1,2,4-triazines **4** also exhibited inhibitory activities against glycogen synthase kinase 3 (GSK3β), and may therefore be developed for the treatment of haematological diseases, and inhibition of phosphodiesterase 10 (PDE10), which is potentially useful for the treatment of neurodegenerative diseases, especially Parkinson’s disease [11].

The scaffolds **1**–**4** consist of a 1,2,4-triazine core, which arises from the ring fusion of C4a–N8 (**1**, **3** and **4**) or C8a–N5 atoms (**2**), and differ in the arrangement of the substitution pattern of the imidazotriazine framework (Figure 1).

The structural variation of imidazo[1,2,4]triazine derivatives poses a significant challenge, particularly if a broad variety of substituents is to be introduced. Several synthetic strategies involving combinatorial and sequential approaches, in particular intramolecular cyclocondensation reactions of functionalized 1,2,4-triazole and imidazole precursors, have been developed [5–6,12].

We have been interested in expanding the medicinal-chemical space of synthetic drug-like small molecules focusing on 6,5-heterobicyclic ring systems in order to increase the diversity of our proprietary compound library [13]. Criteria for selection of the target structures include the potential for biological activity and bioavailability (peroral, and possibly central nervous system), structural novelty, synthetic accessibility, and the possibility for broad structural variations. In particular, we planned to introduce sp^3^-hybridized carbon atoms, to avoid completely flat aromatic structures that are prone to low solubility in water due to π-stacking effects.

**Scheme 1:**
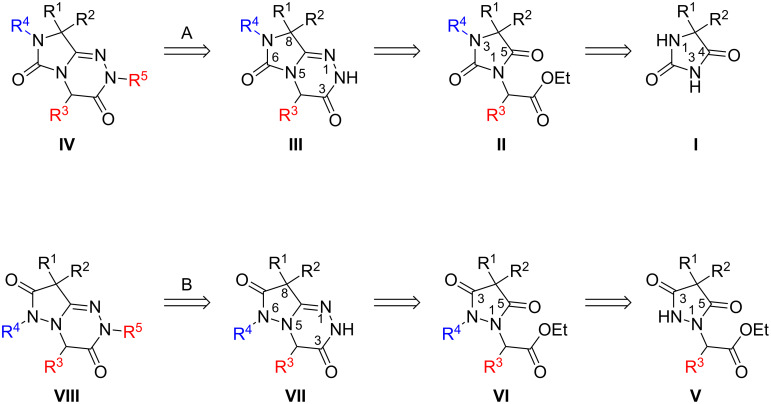
Retrosynthetic approaches towards novel 7,8-dihydroimidazo-[5,1-*c*][1,2,4]-triazine-3,6-diones **IV** and 6,8-dihydropyrazolo[5,1-*c*][1,2,4]triazine-3,7-diones **VIII**.

We therefore identified the 1,2,4-triazine-containing scaffolds **IV** and **VIII** as promising novel target structures (Scheme 1). Depending on the starting material used, e.g., hydantoin **I**, or pyrazolidine-3,5-dione **V**, respectively, either 7,8-dihydroimidazo[5,1-*c*][1,2,4]triazine-3,6-diones **IV** (route A) or 6,8-dihydropyrazolo[5,1-*c*][1,2,4]triazine-3,7-dione derivatives **VIII** (route B) should be accessible. In either case two nitrogen atoms, N7/N6 and N2, may be substituted with a variety of residues; e.g., alkylation of N2 (R^5^) in the very last step should allow an easy access to a library of compounds. Route B would proceed in four steps yielding product **VIII** starting from **V**, which may be obtained from the corresponding malonyl dichloride and hydrazine derivatives.

In the present study we focussed on synthetic route A starting from the commercially available hydantoins **I**, which would allow very broad structural diversity by introducing a variety of substituents and functionalities at different stages of the synthesis. As a first step ethyl (2,5-dioxoimidazolidin-1-yl)acetate derivative **II** is formed from hydantoins **I**. The condensation reaction of **II** with hydrazine, followed by a regioselective intramolecular heterocyclization, formally by dehydration, would afford the 7,8-dihydroimidazo[5,1-*c*][1,2,4]triazine-3,6-diones **III**. The success of this route, however, depends on two factors: (i) the preferred regioselectivity of the successive *N*-alkylation steps for the formation of **II**; and (ii) the regioselective cyclization of 2,5-dioxoimidazolidines **II** to the desired product **III**, which is expected to be favoured in the case of dimethylhydantoin derivatives (R^1^ = R^2^ = Me).

## Results and Discussion

### Chemistry

Following the proposed strategy for the formation of 7,8-dihydroimidazo[5,1-*c*][1,2,4]triazine-3,6-dione derivatives possessing aryl (R^4^ = 3,4-dichlorobenzyl) and alkyl/alkynyl (R^5^ = Me, propargyl) substituents at their nitrogen atoms 7 and 2, respectively, we started from the differently substituted hydantoins (R^1^ = R^2^ = H or Me) **5** and **6**. N3- and subsequent N1-alkylation led to the N1,N3-dialkylated hydantoin derivatives **12**–**18**. Alkylation reactions of hydantoins are well documented in the literature [14–18]. Depending on the alkylation reagents and reaction conditions, either N1- or N3-substituted hydantoins are accessible [14–17]. The details of the successfully conducted N3-alkylation of hydantoins **5** and **6** with various alkyl halides are shown in Table 1.

**Table 1:** Yields and reaction conditions for the N3-alkylation of hydantoin derivatives **5** and **6**.

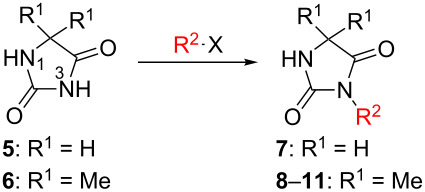				

starting compound	R^2^–X (1.1 equiv)	conditions	product	yield (%)

**5**	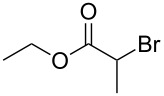	1.1 equiv K_2_CO_3_ DMF, 85 °C, 42 h	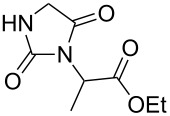 **7**^a^	89
**6**	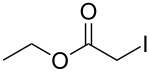	1.1 equiv K_2_CO_3_ DMF, 80 °C, 56 h	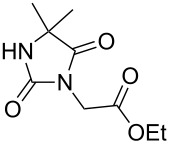 **8**^a^	76
**6**	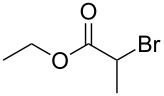	1.1 equiv K_2_CO_3_ DMF, 85 °C, 90 h	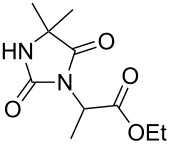 **9**^a^	92
**6**	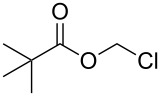 = POM-Cl	1.1 equiv K_2_CO_3_ DMF, 85 °C, 28 h	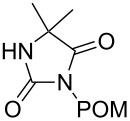 **10**	56
**6**	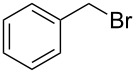	1.1 equiv K_2_CO_3_ DMF, 85 °C, 6 h	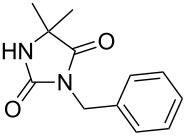 **11**^b^	92

^a^Compounds **7** [19], **8** [19–20], **9** [19] have been described in the literature without detailed analytical data. ^b^Analytical data for **11** [14] are in accordance with literature data.

Alkylation of the thermodynamically preferred N3-position was achieved following a modified literature procedure, and the products could be obtained on a multigram scale [18]. In general, 1.1 equivalents of the corresponding alkylation reagent and potassium carbonate as a base in dry dimethylformamide (DMF) were used. The best yield (92%) was achieved with ethyl 2-bromopropionate and benzyl bromide as alkylating reagents. Although this procedure led to the direct formation of the desired N3-substituted products, the regioselectivity depended on the substitution pattern of **6**, due to the directing effect of the methyl groups. Thus, traces of a N1-substituted regioisomer were detected by LC/ESI-MS analysis in the conversion of **5** to product **7** (N3/N1-alkylation ratio 15:1). Direct N1,N3-dialkylation of **5**, however, was not observed. Alkylation with benzyl bromide was carried out in order to verify the N3-substitution yielding **11**, which had already been described in the literature [14]. 3-Benzyl-5,5-dimethylhydantoin (**11**) was obtained in 92% isolated yield (see Section Experimental in Supporting Information File 1), in comparison to 80% obtained by the literature procedure [14]. Compounds **7**–**11** were isolated after chromatographic purification in good to excellent yields and analyzed by NMR spectroscopy (^1^H and ^13^C) and LC/ESI-MS. In addition, we obtained an X-ray crystal structure of **11** [18], which confirmed the alkylation at the N3-position of 5,5-dimethylhydantoin (**6**). The subsequent N1-alkylation of 2,5-dioxoimidazolidines **7**–**10** to the corresponding *N*,*N*-disubstituted hydantoins **12**–**18** was performed with 1.0–1.2 equiv of the appropriate alkylating reagent by using sodium hydride as a base in dry dimethylformamide (DMF) (Table 2). However, under these conditions the formation of compounds **12**–**18** strongly depended on the nature of the alkylating reagent, and therefore varying yields of dialkylated products were obtained. The reactions of **9** and **10** with 3,4-dichlorobenzyl bromide and phenethyl bromide, respectively, led to the formation of **15**, **16**, and **18** in poor to moderate yields. The alkylation with benzyl bromide worked well not only with sodium hydride (method 1) but also in the presence of potassium carbonate (method 2) as a base and gave rise to the disubstituted derivatives **12**–**14** and **17** in good yields. Products **12**–**18** were isolated after chromatographic purification (Table 2).

**Table 2:** Yields and reaction conditions of N1-alkylation of N3-substituted hydantoins **7**–**10**.

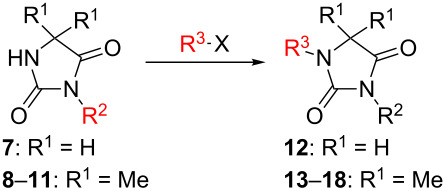				

starting compound	R^3^–X (1.0–1.2 equiv)	conditions	product	yield (%)

**7**	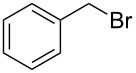	1.2 equiv NaH DMF, 85 °C, 66 h^a^	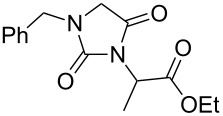 **12**	70
**8**	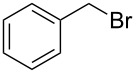	1.0 equiv NaH DMF, 80 °C, 72 h^a^	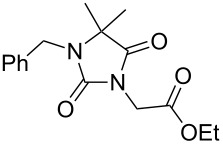 **13**	80
**9**	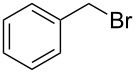	(1) 1.2 equiv NaH^a^ DMF, 85 °C, 80 h	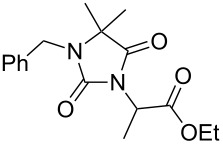 **14**	79^a^
		(2) 1.1 equiv K_2_CO_3_ DMF, 80 °C, 72 h^b^		60^b^
**9**	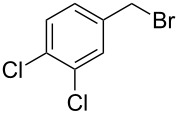	1.2 equiv NaH DMF, 85 °C, 120 h^a^	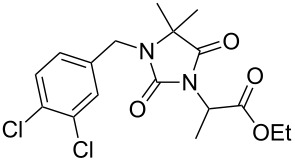 **15**	37
**9**	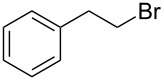	1.1 equiv NaH DMF, 85 °C, 68 h^a^	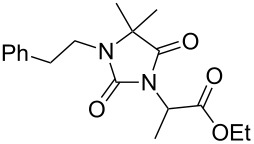 **16**	18
**10**	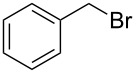	1.2 equiv K_2_CO_3_ DMF, 85 °C, 72 h^b^	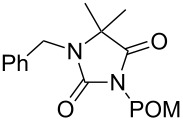 **17**	62
**10**	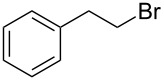	1.2 equiv K_2_CO_3_ DMF, 85 °C, 69 h^b^	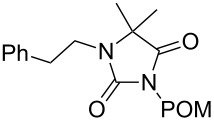 **18**	28

^a^Method 1. ^b^Method 2.

**Scheme 2:**
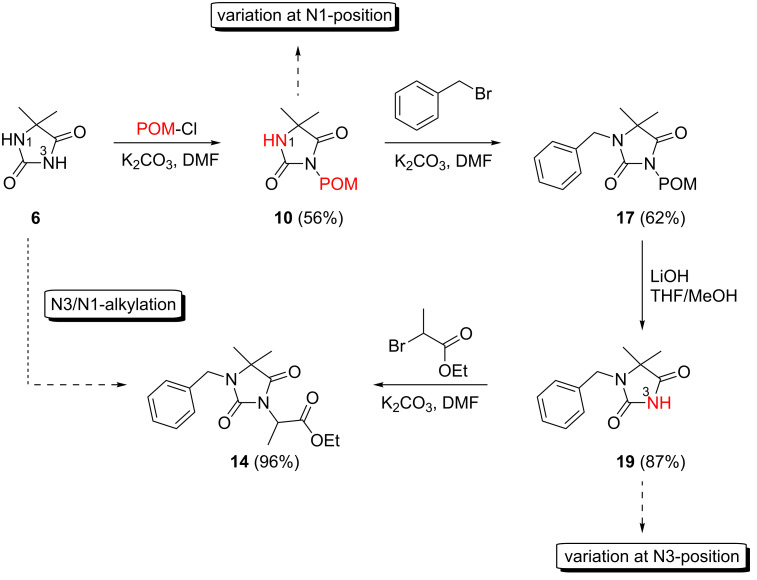
Synthesis of N3-unsubstituted, N1-substituted hydantoin **19** by using a protection strategy.

In order to demonstrate the tractability of the successive N1,N3-alkylation of hydantoin **6** we applied a three-step synthetic route to obtain compound **19** [17]. N3-Unsubstituted, N1-substituted hydantoins were obtained by a three-step synthetic procedure by reaction of **6** with pivaloyloxymethyl chloride (POM-Cl) to introduce a protecting group prior to N1-alkylation. Subsequently, the ester can easily be cleaved with lithium hydroxide in a methanol/tetrahydrofuran mixture at room temperature to afford 1-benzyl-5,5-dimethylimidazolidine-2,4-dione (**19**) (Scheme 2).

**Scheme 3:**
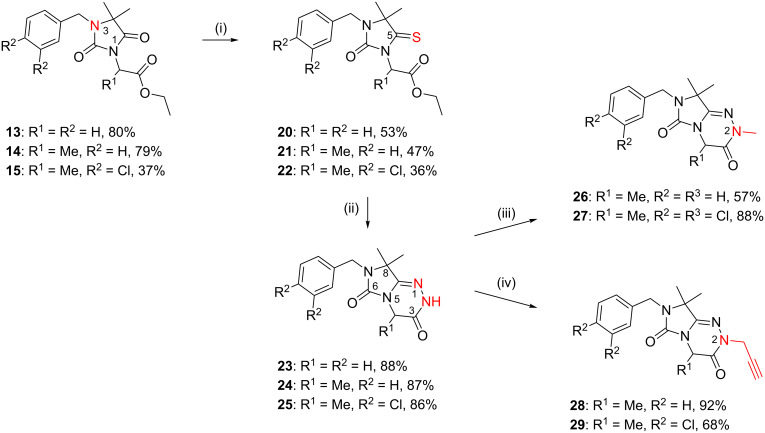
Synthesis of 7,8-dihydroimidazo[5,1-*c*][1,2,4]triazine-3,6-diones **23**–**29**. Reagents and conditions: (i) P_2_S_5_, dioxane, reflux, 24 h; (ii) hydrazine monohydrate (20 equiv), EtOH, reflux 5–10 h; (iii) MeI (for **26**) or methyl methanesulfonate (for **27**), NaH, DMF, r.t.; (iv) propargyl bromide (80% in toluene), NaH, 85 °C, 48 h.

According to our retrosynthetic analysis (Scheme 1), and based on an efficient protocol for the regioselective *N*-alkylation of hydantoins, we applied a four-step procedure for the synthesis of differently substituted 7,8-dihydroimidazo[5,1-*c*][1,2,4]triazine-3,6-diones **23**–**29** (Scheme 3).

**Scheme 4:**
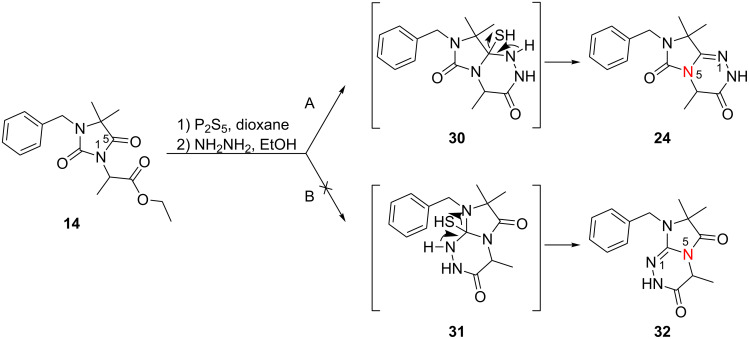
Proposed regioselective two-step cyclization pathway to form **24** from **14**.

The main challenge in the synthesis of **23**–**25** was the construction of the 1,2,4-triazine ring. Direct reaction of **13**–**15** with hydrazine hydrate failed, and only the corresponding hydrazides were formed. Therefore, the C5 carbonyl group of **13**–**15** was regioselectively thionated with phosphorus pentasulfide in dioxane under reflux [21] (Scheme 4). The products were purified chromatographically and the preferred regioselectivity (C5 versus C2) was confirmed by NMR spectroscopy. The formation of C2-thiocarbonyl by-products was not observed under these reaction conditions. The highly regioselective formation of thioketones **20**–**22** may be due to electronic effects and prevention of the formation of certain tautomers by the C4-methyl groups in compounds **13**–**15** [22]. To investigate the influence of the C4-methyl groups on the regioselective thionation reaction, we treated unsubstituted 5,5-dimethylhydantoin **6** with phosphorus pentasulfide in dioxane for four hours. As reported in the literature, we observed only the preferred C4 thiocarbonyl product while the thionation of 5-unsubstituted hydantoin yields a mixture of 2- and 4-thionated products, indicating that the 5-dimethyl substitution was responsible for regioselective thionation [21].

The proposed intramolecular N1–C5 heterocondensation as a key step to form the desired imidazo[5,1-*c*][1,2,4]triazine-3,6-diones **23**–**25** was accomplished by reaction of the thioxoimidazolidines **20**–**22** with hydrazine hydrate [23–26]. Different reaction conditions were applied, e.g., variation of the solvent or the amount of hydrazine monohydrate that was used. The highest yields of compounds **23**–**25** (almost 90% on average) were obtained when the condensation was performed in ethanol with a large excess of hydrazine hydrate (20 equiv) under reflux for the appropriate reaction time. We observed that the adding of molecular sieves (4 Å) to the reaction medium greatly improved the yields of condensed products **23**–**25**. The regioselective two-step cyclization of **14** yielding imidazo[5,1-*c*][1,2,4]triazine-3,6-dione (**24**, pathway A) via an N5–C5 ring fusion is outlined in Scheme 4. Precursors **13** and **15** follow the same pathway A. A dehydrothionated intermediate (e.g., **30** in the preparation of **24**) is formed. Obviously, N1–C2 cyclization (pathway B) via **31** is not favoured, and **32** is not formed starting from **21**. The products **23**–**25** were purified by column chromatography and obtained on a multigram scale. Finally, the imidazotriazines were further functionalized by an N-alkylation reaction using different alkylating reagents under basic conditions (Scheme 3). Alkylation of **24** with methyl iodide led to **26**, whereas methyl methanesufonate was used as an alkylating reagent for the methylation of **25** yielding **27**. Reaction of **24** and **25** with propargyl bromide (80% in toluene) under basic conditions at 85 °C yielded the *N*-propargyl derivatives **28** and **29** (Scheme 3). The final products **26**–**29** were purified by column chromatography followed by preparative RP-HPLC, or by recrystallization; the pure products were obtained in good yields.

### Structural analyses

Regioselectivity of the thionation reaction of the C5-carbonyl group was an essential precondition for a successful heterocondensation step yielding the desired imidazo[5,1-*c*][1,2,4]triazine-3,6-dione derivatives **23**–**25**. The structural assignment of the precursors (2,5-dioxoimidazolidines **13**–**15** and their thiocarbonyl analogues **20**–**22**) reported herein is based on their spectral data and, if necessary, supported by MMFF94 force field conformational analytical data [27]. The most important ^13^C NMR chemical shifts δ (ppm) are reported in Table 3. In the case of C5-thionation, the ^13^C NMR signal for this carbon atom is shifted from about 176 to 208–209 ppm. In addition, the C4 signal is shifted from ca. 62 to 71 ppm upon thionation.

**Table 3:** ^13^C NMR chemical shifts (determined at 125 MHz in DMSO-*d*_6_) before and after thionation: comparison of **13**–**15** and **20**–**22**.

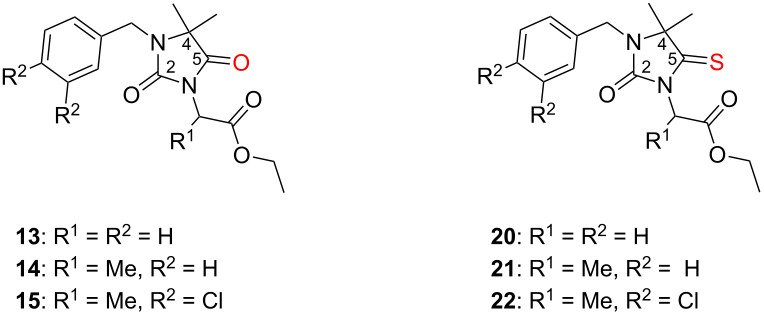						

^13^C assignment	**13**	**14**	**15**	**20**	**21**	**22**

C2	154.6	154.5	154.6	154.1	153.9	154.0
C4	62.2	61.8	61.9	70.9	70.8	70.8
C5	175.9	175.7	175.6	209.2	208.5	208.4

**Figure 2:**
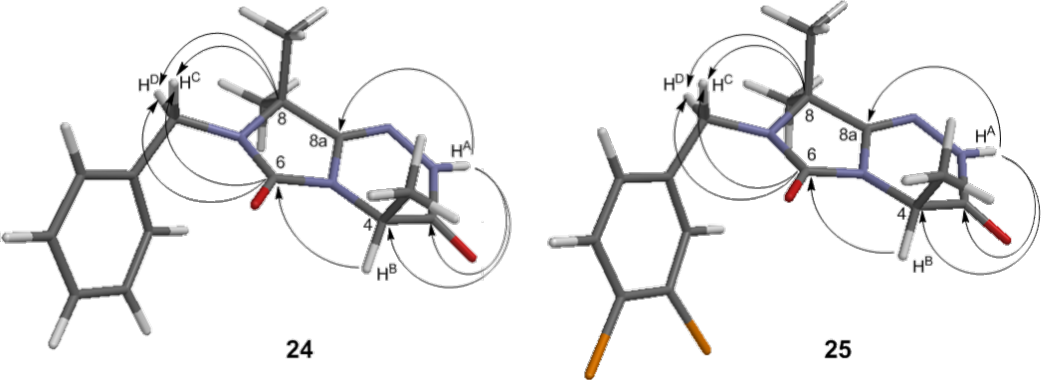
Optimized structure (MMFF95) and key HMBC correlations of imidazo[5,1-*c*][1,2,4]triazine-3,6(2*H*,4*H*)-diones **24** and **25**.

The structure determination of the condensed key products **24** and **25** was carried out by heteronuclear correlation NMR (HSQC and HMBC) in combination with ^1^H and ^13^C NMR, and additionally by LC/ESI-MS (*m*/*z* 287 [M + H]^+^/285 [M − H]^−^ for **24**, 355 [M + H]^+^/353 [M − H]^−^ for **25**). Molecular modelling was performed to calculate the respective geometries by using the MMFF95 force field [27] assuming an N1–C5 ring fusion during the intramolecular condensation reaction yielding **24** and **25** (Figure 2).

**Figure 3:**
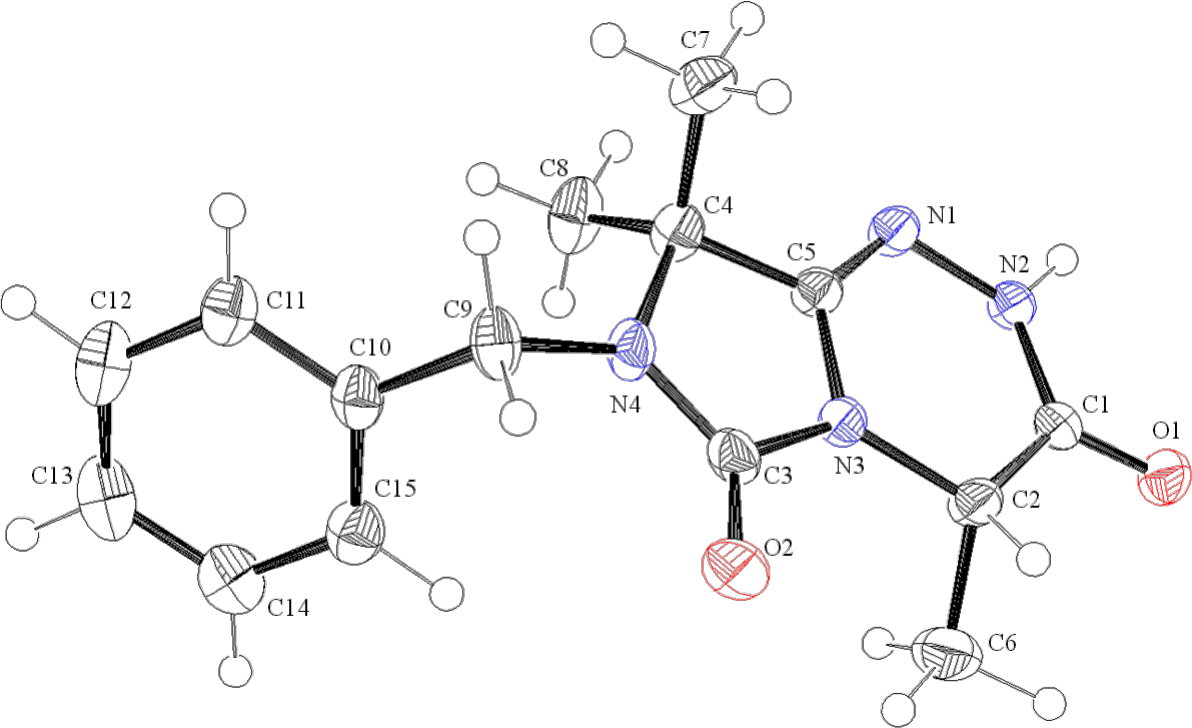
ORTEP diagram of **24** showing the atomic numbering. The thermal ellipsoids are drawn at the 50% probability level.

The structural assignments of **24** and **25** were confirmed by HSQC and, most importantly, by HMBC (see Supporting Information File 1). The analysis of two HMBC spectra of **24** and **25** showed key correlations between C8 and H^C^/H^D^ as well as between C6 and H^C^/H^D^ methylene protons. In the case of an alternative N1–C2 ring fusion (structure **32** in Scheme 4) such a correlation between the carbonyl C5-function and the methylene protons H^C^/H^D^ would be not possible. The structure was additionally confirmed by X-ray analysis of **24** (Figure 3) [28]. The molecules in the crystal are held together by one type of intermolecular hydrogen bond, located between O2 and the hydrogen H1 of the N2, with a distance of *d*(O···H1–N2) = 1.9437(2) Å, *d*(H1–N2) = 0.93(2) Å and an angle of 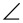
(O2–H1–N2) = 155(2)°.

### Physicochemical properties

In order to assess the physicochemical properties of imidazo[5,1-*c*][1,2,4]triazine-3,6-dione derivatives we determined water-solubility, log *P*, and p*K*_a_ values for compound **25** as a representative of this new class of heterocyclic compounds. Water solubility at physiological pH of 7.4 was found to be 47 µg/mL, which is a suitable range for perorally active drugs. A p*K*_a_ value of 10.0, and a log*P* value of 3 was determined. Thus, the molecule will be uncharged under physiological conditions and the log*P* value is in a range which allows us to predict oral bioavailability [29].

## Conclusion

In conclusion, we have designed and synthesized novel fused 7,8-dihydroimidazo-[5,1-*c*][1,2,4]triazine-3,6(2*H*,4*H*)-dione derivatives, i.e., a novel class of small heterocyclic molecules with drug-like properties, thereby expanding the druggable chemical space. For this purpose, we have developed a four-step convergent synthetic concept to access the imidazo[5,1-*c*][1,2,4]triazine frameworks starting from the commercially available 5,5-dimethylhydantoin **6**. The reaction sequence involves successive *N*-alkylations of the corresponding hydantoin, followed by C5-thionation and an intramolecular heterocondensation reaction with hydrazine hydrate as a key step affording the imidazo[5,1-*c*][1,2,4]triazine-3,6-dione derivatives **23**–**25** in a regioselective manner. The synthetic procedure was optimized for all steps and can easily be carried out on a multigram scale. The experimentally determined physicochemical properties of prototypic compound **25** are indicative of drug-like properties suitable for peroral application. Alkylation in the final step allows the introduction of additional diversity and the preparation of compound libraries. The new synthetic strategy should also allow for the preparation of other, related heterobicyclic systems possessing different ring-members, ring sizes, and a variety of substituents.

## Supporting Information

File 1 Assays for determination of physicochemical properties of **25**, experimental details and copies of NMR (1D and 2D) and LC/ESI-MS spectra of compounds **24** and **25**.

## Dedication

Dedicated to the late Dr. Frédéric Denonne, who inspired this work: to a great medicinal chemist, a fantastic colleague, and a wonderful person who died much too young.
