# Supplementary material for: Regioselective synthesis of 7,8-dihydroimidazo[5,1-c][1,2,4]triazine-3,6(2H,4H)-dione derivatives: A new drug-like heterocyclic scaffold
Source: Beilstein J Org Chem. 2012 Sep 20;8:1584–93. doi: 10.3762/bjoc.8.181 (PMC3510990; doi:10.3762/bjoc.8.181)
Supplement: File 1 — Assays for determination of physicochemical properties of 25, experimental details and copies of NMR (1D and 2D) and LC/ESI-MS spectra of compounds 24 and 25. [file Beilstein_J_Org_Chem-08-1584-s001.pdf]

Supporting Information

for

Regioselective synthesis of 7,8-dihydroimidazo[5,1-*c*][1,2,4]triazine-3,6(2*H*,4*H*)-dione derivatives: A new drug-like heterocyclic scaffold

Nikolay T. Tzvetkov[1], Harald Euler[2] and Christa E. Müller*[1]

Address: [1]PharmaCenter Bonn, Pharmaceutical Sciences Bonn (PSB), University of Bonn, Pharmaceutical Institute, An der Immenburg 4, 53121 Bonn, Germany and [2]University of Bonn, Steinmann-Institute of Mineralogy, Poppelsdorfer Schloss, 53115 Bonn, Germany

Email: Christa E. Müller* - christa.mueller@uni-bonn.de

*Corresponding author

Assays for determination of physicochemical properties of 25, experimental details and copies of NMR (1D and 2D) and LC/ESI-MS spectra of compounds 24 and 25

Contents:

- Physicochemical measurements of compound **25** S2
- Experimental S3
- NMR and LC/ESI-MS spectra of **24** and **25** S21

Physicochemical measurements of compound 25: The potentiometric/pH-metric pK_a, logP/D and solubility (S) measurements were performed on a SiriusT3 physchem system (Sirius Analytical Instruments Ltd., Forest Row, UK) equipped with a Ag/AgCl pH electrode as a double-junction reference for the determination of dissociation constants in a pH range 1.8−12.2. The SiriusT3 features up to six automated dispersers for reagent addition and miniaturized sensors to perform automatic titration in 1.0 mL of solution, a stirrer, an in situ UV probe, and automatic calibration and cleaning procedures. The pK_a, logP/D and water-solubility (S) were calculated by using RefinementPro2™ and CheqSol (Bjerrum) software. For the pK_a and logP/D measurements less than 1.0 mg of solid compounds was used, whereas solubility experiments required higher amounts (1.0−2.0 mg).

HPLC determination of thermodynamic water-solubility (*S*):

The thermodynamic water solubility of **25** was determined by HPLC-UV obtained on an HPLC instrument Agilent 1100 using the following procedure: Dissolving of the compound at a concentration of 1.0 mg/mL in methanol (HPLC quality from JT Baker). Then, 3 µL of the compound solution was injected into a Knauer Vertex Plus C 18 HPLC column (50x4 mm) and elution was performed with a gradient of water/methanol containing 0.25% acetic acid (freshly prepared) from 60:40 up to 10:90 (40-90-40) for 12 min at a flow rate of 1.0 mL/min, starting the gradient after 1 min. UV absorption was detected from 190 to 400 nm using a diode array detector. Imipramine was used as a reference compound (solubility S = 18.2 µg/mL). For the calculation of the thermodynamic solubility two injections were performed.

Experimental

General methods

Commercially available chemicals and solvents were used without further purification unless otherwise noted. The reactions were monitored by thin layer chromatography (TLC) using aluminium sheets coated with silica gel 60 F_{254} (Merck). Column chromatography was carried out on silica gel 0.060-0.200 mm (Acros). Melting points were measured on a Büchi Melting Point B-545 apparatus and are uncorrected. Mass spectra were recorded on an API 2000 mass spectrometer (electron spray ion source, Applied Biosystems, Darmstadt, Germany) coupled with an Agilent 1100 HPLC system using a Phenomenex Luna HPLC C18 column (50 × 2.00 mm, particle size 3 µm). ^{1}H and ^{13}C NMR spectra were recorded on a 500 MHz spectrometer (Bruker Avance). $CDCl_3$ and DMSO-d_6 were used as solvent as indicated below. Chemical shifts (δ) are given in parts per million (ppm) related to that of the solvent. Coupling constants *J* are given in hertz (Hz), and spin multiplicities are given as a singlet (s), doublet (d), triplet (t), quartet (q), multiplet (m), and broad (br). All spectra are recorded at room temperature (r.t.). The purity of the final products was determined by HPLC-UV obtained on an LC-MS instrument (Applied Biosystems API 2000 LC-MS/MS, HPLC Agilent 1100) using a procedure as follows: Dissolving of the compounds at a concentration of 1.0 mg/mL in methanol and if necessary sonicating it to complete dissolution. Then, 10 µL of the substance solution was injected into a Phenomenex Luna C 18 HPLC column and elution was performed with a gradient of water/methanol containing either 2 mM ammonium acetate (chrom. system A) or 2 mM ammonium acetate and 0.1% formic acid (chrom. system B) from 90:10 up to 0:100 for 30 min at a flow rate of 250 µL/min, starting the gradient after 10 min. Purification by RP-HPLC of final products was performed on a Eurospher 100–10

C18 column (250 × 20 mm; flow 10 mL/min) by using Knauer Advanced Scientific Instruments preparative pump 1800/100 coupled with a Smartline 2600 UV diode array detector (90 to 400 nm). Elemental analyses were recorded on a Vario EL V2.4 CHN Elemental Analyzer (Elementar Analysesysteme GmbH).

General procedure A for regioselective N-alkylation of hydantoins 5 and 6

A stirred solution of hydantoin **5**, or **6**, respectively, (1.00 equiv) and potassium carbonate (1.00 equiv) in dry DMF (1–2 mL per 1.00 mmol starting material) was treated with the appropriate alkylating reagent (1.00–1.10 equiv) under an argon atmosphere. The solution was stirred at 85 °C while complete conversion was detected by TLC control (eluent: dichloromethane/methanol, 95:5). After all of the starting material had disappeared, the reaction mixture was diluted with water (10–30 mL), neutralized with 2N HCl and extracted with dichloromethane (3 × 50 mL). The combined organic layers were washed with water (2 × 10 mL), dried over sodium sulfate and evaporated under reduced pressure. The crude products were purified by column chromatography on silica gel and if necessary by recrystallization from dichloromethane/petroleum ether (1:9).

2-(2,5-Dioxoimidazolidin-1-yl)propionic acid ethyl ester (7) [19]

Following general procedure A, hydantoin (**5**; 2.00 g, 0.02 mol) was treated with ethyl 2-bromopropionate (3.98 g, 0.022 mol). The product was obtained after 42 h of stirring at 85 °C, as a yellowish oil: Yield 3.56 g (89%); Chromatography on silica gel (eluent: dichloromethane/methanol, 95:5, R_f = 0.81); ^{1}H NMR (500 MHz, DMSO-d_6, δ/ppm) 1.15 (t, J = 7.25 Hz, 3H, $CH_2C\underline{H}_3$), 1.42 (d, J = 6.93 Hz, 3H, $CHC\underline{H}_3$), 3.95 (q, J = 17.97 Hz, 2H, $C\underline{H}_2$), 4.09 (qdqd, J = 3.46, 6.94, 3.78, 7.25 Hz, 2H, $C\underline{H}_2CH_3$), 4.66 (q, J = 7.25 Hz, 1H, $C\underline{H}CH_3$), 8.14 (s, 1H, NH); ^{13}C NMR (125 MHz, DMSO-d_6,

δ/ppm) 14.08 ($CH\underline{C}H_3$), 14.58 ($CH_2\underline{C}H_3$), 45.93 (CH_2), 47.05 ($\underline{C}HCH_3$), 61.19 ($\underline{C}H_2CH_3$), 156.62 (C2), 169.52 (O=CO), 171.40 (C5); LC/ESI-MS (*m*/*z*): negative mode 199 ($[M-H]^-$), positive mode: not applicable. Purity (HPLC-UV 206 nm): 95.5%.

(4,4-Dimethyl-2,5-dioxoimidazolidin-1-yl)acetic acid ethyl ester (8) [20]

Following general procedure A, 5,5-dimethylhydantoin (**6**; 2.00 g, 0.016 mol) was treated with ethyl iodoacetate (3.67 g, 0.017 mol). The product was obtained after 56 h of stirring at 85 °C as white crystals: Yield 2.55 g (76%); mp 82.7–83.0 °C (dec.); Chromatography on silica gel (eluent: dichloromethane/methanol, 90:10, R_f = 0.77) and recrystallization from dichloromethane/petroleum ether; ^{1}H NMR (500 MHz, DMSO-d_6, δ/ppm) 1.18 (t, *J* = 7.25 Hz, 3H, $CH_2C\underline{H}_3$), 1.30 (s, 6H, $C(C\underline{H}_3)_2$), 4.12 (q, *J* = 7.25 Hz, 2H, $C\underline{H}_2CH_3$), 4.13 (s, 2H, $C\underline{H}_2$), 8.40 (s, 1H, NH); ^{13}C NMR (125 MHz, DMSO-d_6, δ/ppm) 14.06 ($CH_2\underline{C}H_3$), 24.62 ($C(\underline{C}H_3)_2$), 39.06 ($\underline{C}H_2CO_2Et$), 40.62 (C4), 58.27 ($\underline{C}H_2Ph$), 61.33 ($\underline{C}H_2CH_3$), 154.67 (C2), 167.56 (O=CO), 177.05 (C5); LC/ESI-MS (*m*/*z*): negative mode 213 ($[M - H]^-$), positive mode 215 ($[M + H]^+$). Purity (HPLC-UV 208 nm): 97.5%.

2-(4,4-Dimethyl-2,5-dioxoimidazolidin-1-yl)propionic acid ethyl ester (9) [19].

Following general procedure A 5,5-dimethylhydantoin (**6**; 4.00 g, 31.2 mmol) was treated with ethyl 2-bromopropionate (6.22 g, 34.4 mmol). The product was obtained after 90 h of stirring at 85 °C as a yellowish oil: Yield 6.55 g (92%); Chromatography on silica gel (eluent: dichloromethane/methanol, 95:5, R_f = 0.78); ^{1}H NMR (500 MHz, DMSO-d_6, δ/ppm) 1.14 (t, *J* = 7.25 Hz, 3H, $CH_2C\underline{H}_3$), 1.27 (s, 3H, $C(C\underline{H}_3)_2$), 1.28 (s, 3H, $C(C\underline{H}_3)_2$), 1.43 (d, *J* = 7.25 Hz, 3H, $CHC\underline{H}_3$), 4.05 (dqt, *J* = 3.47 / 10.72 / 7.25 Hz, 1H, $C\underline{H}_2CH_3$), 4.11 (dqt, *J* = 3.47 / 10.72 / 7.25 Hz, 1H, $C\underline{H}_2CH_3$), 4.63 (q, *J* = 7.25 Hz, 1H, $C\underline{H}CH_3$), 8.36 (s, 1H, NH); ^{13}C NMR (125 MHz, DMSO-d_6, δ/ppm) 14.04

$(CH\underline{C}H_3)$, 14.52 $(CH_2\underline{C}H_3)$, 24.54 $(C(\underline{C}H_3)_2)$, 24.59 $(C(\underline{C}H_3)_2)$, 47.04 $(\underline{C}HCH_3)$, 57.77 (C4), 61.17 $(\underline{C}H_2CH_3)$, 154.56 (C2), 169.52 $(O{=}\underline{C}O)$, 176.82 (C5); LC/ESI-MS (*m*/*z*): negative mode 227 ($[M - H]^-$), positive mode 229 ($[M + H]^+$). Purity (HPLC-UV 204 nm): 96.9%.

(4,4-Dimethyl-2,5-dioxoimidazolidin-1-yl)methyl pivalate (10).

Following general procedure A, 5,5-dimethylhydantoin (**6**; 5.00 g, 0.039 mol) was treated with pivaloyloxymethyl chloride (POM-Cl, 5.90 g, 0.039 mol). The product was obtained after 28 h of stirring at 85 °C as a white solid: Yield 5.32 g (56%); mp 80.7–81.3 °C (dec.); Chromatography on silica gel (eluent: dichloromethane/methanol, 95:5, R_f = 0.67) and recrystallization from dichloromethane/petroleum ether; ^{1}H NMR (500 MHz, DMSO-d_6, δ/ppm) 1.10 (s, 9H, $C(C\underline{H}_3)_3$), 1.29 (s, 6H, $C(C\underline{H}_3)_2$), 5.34 (s, 2H, $C\underline{H}_2$), 8.48 (s, 1H, NH); ^{13}C NMR (125 MHz, DMSO-d_6, δ/ppm) 24.59 $(C(\underline{C}H_3)_2)$, 26.71 $(C(\underline{C}H_3)_3)$, 38.38 $(\underline{C}(CH_3)_3)$, 58.03 (C4), 61.61 (CH_2), 153.61 (C2), 176.48 $(O{=}\underline{C}C(CH_3)_3)$, 176.65 (C5); LC/ESI-MS (*m*/*z*): negative mode 241 ($[M - H]^-$), positive mode 243 ($[M + H]^+$). Purity (HPLC-UV 200 nm): 97.8%.

3-Benzyl-5,5-dimethylimidazolidine-2,4-dione (11)

The product was obtained according to the literature as white crystals [18]: Yield 1.56 g (92%, Lit. 80%) [14]; mp 104.4–105.1 °C (dec.).

General procedure B for N-alkylation of hydantoin derivatives 7–10

Method 1: A suspension of sodium hydride (60% in paraffin) was washed under an argon atmosphere with *n*-hexane (3 × 10 mL), and the solvent was removed under reduced pressure. The remaining material (1.20 equiv) of sodium hydride was suspended under an argon atmosphere in anhydrous DMF or THF (1–2 mL per 1.00 mmol starting material), stirred for 10 min at room temperature, and treated with the corresponding *N*-alkylated hydantoins **7–10** (1.00 equiv). The reaction mixture was stirred at room temperature for 15 min until hydrogen evolution started, and a solution of the appropriate alkylating reagent (1.10–1.20 equiv) in anhydrous DMF or THF (0.25–0.30 mL per 1.00 mmol alkylating reagent) was added dropwise. The solution was stirred at 80–85 °C while complete conversion was detected by TLC control (eluent: dichloromethane/methanol, 95:5). After all of the starting material had disappeared, the reaction mixture was diluted with water (10–30 mL), neutralized with 2N HCl and extracted with dichloromethane (3 × 50 mL). The combined organic layers were washed with water (2 × 10 mL), dried over sodium sulfate, filtered and evaporated under reduced pressure. The crude products were purified by column chromatography on silica gel.

Method 2: To a well-stirred solution of the corresponding *N*-alkylated hydantoin (1.00 equiv) and potassium carbonate (1.20 equiv) in dry DMF (1–2 mL per 1.00 mmol starting material) was treated with the appropriate alkylating reagent (1.00–1.10 equiv) under argon atmosphere. The solution was stirred at 80–85 °C while complete conversion was detected by TLC control (eluent: dichloromethane/methanol, 95:5). The final products were isolated after aqueous work-up and the purification procedure described for method 1.

2-(3-Benzyl-2,5-dioxoimidazolidin-1-yl)propionic acid ethyl ester (12).

Following general procedure B (Method 1 in DMF), 2-(4,4-dimethyl-2,5-dioxoimidazolidin-1-yl)propionic acid ethyl ester (**7**; 1.70 g, 8.49 mmol) was treated with benzyl bromide (1.74 g, 10.2 mmol). The product was obtained after 66 h of stirring at 85 °C, as a yellowish oil: Yield 1.71 g (70%); Chromatography on silica gel (eluent: dichloromethane/methanol, 95:5, R_f = 0.98); ^{1}H NMR (500 MHz, DMSO-d_6, δ/ppm) 1.15 (t, J = 6.93 Hz, 3H, $CH_2C\underline{H}_3$), 1.46 (d, J = 7.25 Hz, 3H, $CHC\underline{H}_3$), 3.94 (s, 2H, $C\underline{H}_2$), 4.09 (qd, J = 3.46 / 6.93 Hz, 1H, $C\underline{H}_2CH_3$), 4.12 (qd, J = 3.79 / 7.25 Hz, 1H, $C\underline{H}_2CH_3$), 4.52 (q, J = 15.45 Hz, 2H, $C\underline{H}_2Ph$), 4.73 (q, J = 7.25 Hz, 1H, $C\underline{H}CH_3$), 7.28–7.38 (m, 5H, Ar); ^{13}C NMR (125 MHz, DMSO-d_6, δ/ppm) 14.09 ($CH\underline{C}H_3$), 14.60 ($CH_2\underline{C}H_3$), 45.84 (C4), 47.68 ($\underline{C}HCH_3$), 49.37 ($\underline{C}H_2Ph$), 61.36 ($\underline{C}H_2CH_3$), 127.68 (Ar), 127.76 (2×C, Ar), 128.89 (2×C, Ar), 136.42 (Ar), 155.67 (C2), 169.47 (C5), 169.56 (O=$\underline{C}$O); LC/ESI-MS (m/z): positive mode 319 ($[M + H]^+$). Purity (HPLC-UV 200 nm): 95.6%.

2-(3-Benzyl-4,4-dimethyl-2,5-dioxoimidazolidin-1-yl)acetic acid ethyl ester (13).

Following general procedure B (Method 1 in DMF) (4,4-Dimethyl-2,5-dioxoimidazolidin-1-yl)acetic acid ethyl ester (**8**; 1.36 g, 6.35 mmol) was treated with benzyl bromide (1.30 g, 7.60 mmol). The product was obtained after 72 h of stirring at 80 °C, as a colourless oil: Yield 1.55 g (80%); Chromatography on silica gel (eluent: dichloromethane/methanol, 95:5, R_f = 0.91); ^{1}H NMR (500 MHz, DMSO-d_6, δ/ppm) 1.19 (t, J = 7.25 Hz, 3H, $CH_2C\underline{H}_3$), 1.24 (s, 6H, $C(C\underline{H}_3)_2$), 4.14 (q, J = 6.93 Hz, 1H, $C\underline{H}_2CH_3$), 4.23 (s, 2H, $C\underline{H}_2CO_2Et$), 4.54 (s, 2H, $C\underline{H}_2Ph$), 7.23–7.27 (m, 1H, Ar), 7.32–7.35 (m, 4H, Ar); ^{13}C NMR (125 MHz, DMSO-d_6, δ/ppm) 14.04 ($CH_2\underline{C}H_3$), 22.67 ($C(\underline{C}H_3)_2$), 39.06 ($\underline{C}H_2CO_2Et$), 42.28 ($\underline{C}H_2Ph$), 61.44 ($\underline{C}H_2CH_3$), 62.18 (C4), 127.34

(Ar), 127.51 (2×C, Ar), 128.50 (2×C, Ar), 138.41 (Ar), 154.58 (C2), 167.46 (O=$\underline{C}$O), 175.92 (C5); LC/ESI-MS (*m*/*z*): positive mode 305 ($[M + H]^+$). Purity (HPLC-UV 212 nm): 96.6%.

2-(3-Benzyl-4,4-dimethyl-2,5-dioxoimidazolidin-1-yl)propionic acid ethyl ester (14).

Following general procedure B (Method 1 in DMF) 2-(4,4-dimethyl-2,5-dioxoimidazolidin-1-yl)propionic acid ethyl ester (**9**; 6.50 g, 28.5 mmol) was treated with benzyl bromide (5.84 g, 34.1 mmol). The product was obtained after 80 h of stirring at 85 °C as a yellowish oil: Yield 7.15 g (79%); Chromatography on silica gel (eluent: dichloromethane/methanol, 90:10, R_f = 0.96); ^{1}H NMR (500 MHz, DMSO-d_6, δ/ppm) 1.14 (t, *J* = 7.25 Hz, 3H, $CH_2C\underline{H}_3$), 1.21 (s, 3H, $C(C\underline{H}_3)_2$), 1.24 (s, 3H, $C(C\underline{H}_3)_2$), 1.49 (d, *J* = 7.26 Hz, 3H, $CHC\underline{H}_3$), 4.08 (dqt, *J* = 3.47 / 10.72 / 7.25 Hz, 1H, $C\underline{H}_2CH_3$), 4.14 (dqt, *J* = 3.47 / 10.72 / 7.25 Hz, 1H, $C\underline{H}_2CH_3$), 4.52 (q, *J* = 16.08 Hz, 2H, $C\underline{H}_2Ph$), 4.75 (q, *J* = 7.25 Hz, 1H, $C\underline{H}CH_3$), 7.24–7.28 (m, 1H, Ar), 7.33 (d, *J* = 0.94 Hz, 2H, Ar), 7.34 (dd, *J* = 0.94 / 1.89 Hz, 2H, Ar); ^{13}C NMR (125 MHz, DMSO-d_6, δ/ppm) 13.99 ($CH\underline{C}H_3$), 14.55 ($CH_2\underline{C}H_3$), 22.61 ($C(\underline{C}H_3)_2$), 22.68 ($C(\underline{C}H_3)_2$), 42.28 ($\underline{C}H_2Ph$), 47.61 ($\underline{C}HCH_3$), 61.31 ($\underline{C}H_2CH_3$), 61.75 (C4), 127.35 (Ar), 127.53 (2×C, Ar), 128.50 (2×C, Ar), 138.45 (Ar), 154.46 (C2), 169.41 (O=$\underline{C}$O), 175.73 (C5); LC/ESI-MS (*m*/*z*): positive mode 319 ($[M + H]^+$). Purity (HPLC-UV 200 nm): 95.6%.

2-[3-(3,4-Dichlorobenzyl)-4,4-dimethyl-2,5-dioxoimidazolidin-1-yl]propionic acid ethyl ester (15).

Following general procedure B (Method 1 in DMF) 2-(4,4-dimethyl-2,5-dioxoimidazolidin-1-yl)propionic acid ethyl ester (**9**; 4.22 g, 18.49 mmol) was treated with 3,4-dichlorobenzyl bromide (5.32 g, 22.17 mmol). The product was obtained

after 120 h of stirring at 85 °C as a yellowish oil: Yield 2.64 g (37%); Chromatography on silica gel (eluent: dichloromethane/methanol, 95:5, R_f = 0.91); ^{1}H NMR (500 MHz, DMSO-d_6, δ/ppm) 1.14 (t, J = 7.25 Hz, 3H, $CH_2C\underline{H}_3$), 1.24 (s, 3H, CCH_3), 1.27 (s, 3H, CCH_3), 1.49 (d, J = 7.26 Hz, 3H, $CHC\underline{H}_3$), 4.07 (dqt, J = 3.47 / 10.72 / 7.25 Hz, 1H, $C\underline{H}_2CH_3$), 4.13 (dqt, J = 3.47 / 10.72 / 7.25 Hz, 1H, $C\underline{H}_2CH_3$), 4.53 (q, J = 16.4 Hz, 2H, $C\underline{H}_2Ph$), 4.75 (q, J = 7.25 Hz, 1H, $C\underline{H}CH_3$), 7.33 (dd, J = 1.89 / 8.36 Hz, 1H, Ar), 7.60 (s, 1H, Ar), 7.61 (d, J = 5.68 Hz, 1H, Ar); ^{13}C NMR (125 MHz, DMSO-d_6, δ/ppm) 14.01 ($CH\underline{C}H_3$), 14.50 ($CH_2\underline{C}H_3$), 22.50 ($C(\underline{C}H_3)_2$), 22.60 ($C(\underline{C}H_3)_2$), 41.19 ($\underline{C}H_2Ph$), 47.68 ($\underline{C}HCH_3$), 61.35 ($\underline{C}H_2CH_3$), 61.87 (C4), 127.97 (Ar), 129.65 (2×C, Ar), 130.77 (2×C, Ar), 139.83 (Ar), 154.60 (C2), 169.37 (O=$\underline{C}$O), 175.61 (C5); LC/ESI-MS (*m*/*z*): positive mode 387 ($[M + H]^+$). Purity (HPLC-UV 200 nm): 94.6%.

2-(4,4-Dimethyl-2,5-dioxo-3-phenethylimidazolidin-1-yl)propionic acid ethyl ester (16).

Following general procedure B (Method 1 in THF), 2-(4,4-dimethyl-2,5-dioxoimidazolidin-1-yl)propionic acid ethyl ester (**9**; 951 mg, 4.17 mmol) was treated with (2-bromoethyl)benzene (848 mg, 4.58 mmol). The product was obtained after 68 h of stirring at 85 °C, as a white solid: Yield 250 mg (18%); mp 109.5–110.4 °C (dec.); Chromatography on silica gel (eluent: dichloromethane/methanol, 95:5, R_f = 0.98) and recrystallization from dichloromethane/petroleum ether; ^{1}H NMR (500 MHz, DMSO-d_6, δ/ppm) 1.14 (t, J = 7.25 Hz, 3H, $CH_2C\underline{H}_3$), 1.18 (s, 3H, $C(C\underline{H}_3)_2$), 1.19 (s, 3H, $C(C\underline{H}_3)_2$), 1.46 (d, J = 7.25 Hz, 3H, $CHC\underline{H}_3$), 2.90 (dt, J = 2.53 / 8.20 Hz, 2H, $CH_2C\underline{H}_2Ph$), 3.43 (d, J = 8.20 Hz, 2H, $C\underline{H}_2CH_2Ph$), 3.45 (d, J = 8.20 Hz, 1H, $C\underline{H}_2CH_2Ph$), 4.07 (dqt, J = 3.47 / 10.72 / 7.25 Hz, 1H, $C\underline{H}_2CH_3$), 4.13 (dqt, J = 3.47 / 10.72 / 7.25 Hz, 1H, $C\underline{H}_2CH_3$), 4.70 (q, J = 7.25 Hz, 1H, $C\underline{H}CH_3$), 7.21 (tt, J = 1.57 / 1.89 Hz, 1H, Ar), 7.23–7.26 (m, 2H, Ar), 7.27–7.31 (m, *2*H, Ar); ^{13}C NMR (125 MHz,

DMSO-d_6, δ/ppm) 14.04 ($CH\underline{C}H_3$), 14.58 ($CH_2\underline{C}H_3$), 22.18 ($C(\underline{C}H_3)_2$), 22.22 ($C(\underline{C}H_3)_2$), 34.71 ($\underline{C}H_2$), 41.14 ($\underline{C}H_2$), 47.37 ($\underline{C}HCH_3$), 61.23 ($\underline{C}H_2CH_3$), 61.46 (C4), 126.49 (Ar), 128.45 (2×C, Ar), 128.99 (2×C, Ar), 138.91 (Ar), 154.00 (C2), 169.38 ($O{=}\underline{C}O$), 175.67 (C5); LC/ESI-MS (*m*/*z*): positive mode 333 ($[M + H]^+$). Purity (HPLC-UV 200 nm): 98.0%.

(3-Benzyl-4,4-dimethyl-2,5-dioxoimidazolidin-1-yl)methyl pivalate (17). Following general procedure B (Method 2) (4,4-dimethyl-2,5-dioxoimidazolidin-1-yl)methyl pivalate (**10**; 1.89 g, 7.80 mmol) was treated with benzyl bromide (1.33 g, 7.80 mmol). The product was obtained after 72 h of stirring at 85 °C as a light yellow oil: Yield 1.62 g (62%); Chromatography on silica gel (eluent: dichloromethane/methanol, 95:5, R_f = 0.95); ^{1}H NMR (500 MHz, DMSO-d_6, δ/ppm) 1.11 (s, 9H, $C(C\underline{H}_3)_3$), 1.18 (s, 6H, $C(C\underline{H}_3)_2$), 4.61 (t, J = 6.94 Hz, 2H, $C\underline{H}_2$), 5.94 (s, 2H, $OC\underline{H}_2$), 7.20-7.33 (m, 5H, ArH); ^{13}C NMR (125 MHz, DMSO-d_6, δ/ppm) 22.85 ($C(\underline{C}H_3)_2$), 26.78 ($C(\underline{C}H_3)_3$), 34.56 ($\underline{C}(CH_3)_3$), 41.35 ($\underline{C}H_2$), 61.77 (C4), 61.81 ($O\underline{C}H_2$), 125.41 (Ar), 128.24 (2×C, Ar), 128.78 (2×C, Ar), 136.41 (Ar), 153.61 (C2), 176.18 ($O{=}\underline{C}C(CH_3)_3$), 176.65 (C5); LC/ESI-MS (*m*/*z*): positive mode 333 ($[M + H]^+$). Purity (HPLC-UV 210 nm): 94.8%.

(4,4-Dimethyl-2,5-dioxo-3-phenethylimidazolidin-1-yl)methyl pivalate (18). Following general procedure B (method 2) (4,4-dimethyl-2,5-dioxoimidazolidin-1-yl)methyl pivalate (**10**; 1.89 g, 7.80 mmol) was treated with 2-bromoethylbenzene (1.72 g, 8.58 mmol). The product was obtained after 69 h of stirring at 85 °C, as a colourless oil: Yield 760 mg (28%); chromatography on silica gel (eluent: dichloromethane/methanol, 95:5, R_f = 0.98); ^{1}H NMR (500 MHz, DMSO-d_6, δ/ppm) 1.11 (s, 9H, $C(C\underline{H}_3)_3$), 1.17 (s, 6H, $C(C\underline{H}_3)_2$), 2.91 (t, J = 6.94 Hz, 2H, $C\underline{H}_2$), 3.45 (t, J

= 7.25 Hz, 2H, $C\underline{H}_2$), 5.39 (s, 2H, $OC\underline{H}_2$), 7.18-7.30 (m, 5H, ArH); ^{13}C NMR (125 MHz, DMSO-d_6, δ/ppm) 22.14 ($C(\underline{C}H_3)_2$), 26.70 ($C(\underline{C}H_3)_3$), 34.46 (CH_2), 39.12 ($\underline{C}(CH_3)_3$), 41.35 ($\underline{C}H_2$), 61.67 (C4), 61.72 ($O\underline{C}H_2$), 126.50 (Ar), 128.36 (2×C, Ar), 128.42 (2×C, Ar), 138.88 (Ar), 153.16 (C2), 175.46 ($O{=}\underline{C}C(CH_3)_3$), 176.43 (C5); LC/ESI-MS (*m*/*z*): positive mode 473 ($[M + H]^+$). Purity (HPLC-UV 210 nm): 95.7%.

General procedure C for regioselective C5-thionation of 2,5-dioxoimidazolidines 13–15

A well-stirred solution of the corresponding N1,N3-dialkylated dydantoins **13**–**15** (1.00 equiv) and phosphorus pentasulfide (1.10 equiv) in dioxane (2–3 mL per 1.00 mmol starting material) was heated under reflux for 24 h while complete conversion was detected by TLC control (eluent: dichloromethane/methanol, 95:5). After all of the starting material had disappeared, the cooled reaction mixture was diluted with ethanol/water mixture (1:1, 10–30 mL), neutralized with 2*N* HCl and extracted with dichloromethane (3 × 50 mL). The combined organic layers were washed with water (2 × 10 mL), dried over sodium sulfate, filtered and evaporated under reduced pressure. The crude products were purified by column chromatography on silica gel.

2-(3-Benzyl-4,4-dimethyl-2-oxo-5-thioxoimidazolidin-1-yl)acetic acid ethyl ester (20).

Following general procedure C, 2-(3-benzyl-4,4-dimethyl-2,5-dioxoimidazolidin-1-yl)acetic acid ethyl ester (**13**; 845 mg, 2.78 mmol) was treated with phosphorus pentasulfide (1.36 g, 3.05 mmol). The product was obtained as a yellowish oil: Yield 470 mg (53%); chromatography on silica gel (eluent: dichloromethane/methanol, 95:5, R_f = 0.39); ^{1}H NMR (500 MHz, DMSO-d_6, δ/ppm) 1.19 (t, J = 7.25 Hz, 3H,

$CH_2C\underline{H}_3$), 1.36 (s, 6H, $C(C\underline{H}_3)_2$), 4.14 (q, *J* = 6.94 Hz, 1H, $C\underline{H}_2CH_3$), 4.60 (s, 2H, $C\underline{H}_2CO_2Et$), 4.64 (s, 2H, $C\underline{H}_2Ph$), 7.24–7.29 (m, 1H, Ar), 7.30–7.37 (m, 4H, Ar); ^{13}C NMR (125 MHz, DMSO-d_6, δ/ppm) 14.04 ($CH_2\underline{C}H_3$), 26.55 ($C(\underline{C}H_3)_2$), 43.53 ($\underline{C}H_2CO_2Et$), 43.71 ($\underline{C}H_2Ph$), 61.50 ($\underline{C}H_2CH_3$), 70.89 (C4), 127.46 (Ar), 127.50 (2×C, Ar), 128.54 (2×C, Ar), 137.94 (Ar), 154.13 (C2), 166.20 (O=$\underline{C}$O), 209.23 ($\underline{C}$=S); LC/ESI-MS (*m*/*z*): positive mode 321 ($[M+H]^+$). Purity (HPLC-UV 230 nm): 97.6%.

2-(3-Benzyl-4,4-dimethyl-2-oxo-5-thioxoimidazolidin-1-yl)propionic acid ethyl ester (21).

Following general procedure C, 2-(3-benzyl-4,4-dimethyl-2,5-dioxoimidazolidin-1-yl)propionic acid ethyl ester (**14**; 6.78 g, 21.3 mmol) was treated with phosphorus pentasulfide (10.42 g, 23.4 mmol). The product was obtained as a yellowish oil: Yield 3.35 g (47%); chromatography on silica gel (eluent: dichloromethane/methanol, 95:5, R_f = 0.48); ^1H NMR (500 MHz, DMSO-d_6, δ/ppm) 1.13 (t, *J* = 7.25 Hz, 3H, $CH_2C\underline{H}_3$), 1.31 (s, 3H, $C(C\underline{H}_3)_2$), 1.37 (s, 3H, $C(C\underline{H}_3)_2$), 1.54 (d, *J* = 7.26 Hz, 3H, $CHC\underline{H}_3$), 4.07 (dqt, *J* = 3.47 / 10.72 / 7.25 Hz, 1H, $C\underline{H}_2CH_3$), 4.13 (dqt, *J* = 3.47 / 10.72 / 7.25 Hz, 1H, $C\underline{H}_2CH_3$), 4.62 (q, *J* = 16.08 Hz, 2H, $C\underline{H}_2Ph$), 5.35 (q, *J* = 7.25 Hz, 1H, $C\underline{H}CH_3$), 7.25–7.30 (m, 1H, Ar), 7.33 (d, *J* = 0.94 Hz, 2H, Ar), 7.34 (dd, *J* = 0.94 / 1.89 Hz, 2H, Ar); ^{13}C NMR (125 MHz, DMSO-d_6, δ/ppm) 13.58 ($CH\underline{C}H_3$), 13.97 ($CH_2\underline{C}H_3$), 26.50 ($C(\underline{C}H_3)_2$), 26.78 ($C(\underline{C}H_3)_2$), 43.47 ($\underline{C}H_2Ph$), 51.35 ($\underline{C}HCH_3$), 61.33 ($\underline{C}H_2CH_3$), 70.76 (C4), 127.50 (3×C, Ar), 128.57 (2×C, Ar), 137.96 (Ar), 153.90 (C2), 168.41 (O=$\underline{C}$O), 208.47 ($\underline{C}$=S); LC/ESI-MS (*m*/*z*): positive mode 335 ($[M + H]^+$). Purity (HPLC-UV 280 nm): 96.9%.

2-[3-(3,4-Dichlorobenzyl)-4,4-dimethyl-2-oxo-5-thioxoimidazolidin-1-yl]propionic acid ethyl ester (22).

Following general procedure C 2-[3-(3,4-dichlorobenzyl)-4,4-dimethyl-2,5-dioxoimidazolidin-1-yl]propionic acid ethyl ester (**15**; 1.76 g, 4.54 mmol) was treated with phosphorus pentasulfide (2.22 g, 5.00 mmol). The product was obtained as a yellowish oil: Yield 661 mg (36%); chromatography on silica gel (eluent: dichloromethane/methanol, 95:5, R_f = 0.68); ^{1}H NMR (500 MHz, DMSO-d_6, δ/ppm) 1.12 (t, J = 7.25 Hz, 3H, $CH_2C\underline{H}_3$), 1.34 (s, 3H, $C(C\underline{H}_3)_2$), 1.40 (s, 3H, $C(C\underline{H}_3)_2$), 1.54 (d, J = 7.25 Hz, 3H, $CHC\underline{H}_3$), 4.07 (dqt, J = 3.47 / 10.72 / 7.25 Hz, 1H, $C\underline{H}_2CH_3$), 4.13 (dqt, J = 3.47 / 10.72 / 7.25 Hz, 1H, $C\underline{H}_2CH_3$), 4.63 (q, J = 16.4 Hz, 2H, $C\underline{H}_2Ph$), 5.35 (q, J = 7.25 Hz, 1H, $C\underline{H}CH_3$), 7.33 (dd, J = 1.89 / 8.51 Hz, 1H, Ar), 7.61 (s, 1H, Ar), 7.62 (d, J = 5.68 Hz, 1H, Ar); ^{13}C NMR (125 MHz, DMSO-d_6, δ/ppm) 13.54 ($CH\underline{C}H_3$), 13.98 ($CH_2\underline{C}H_3$), 26.35 ($C(\underline{C}H_3)_2$), 26.68 ($C(\underline{C}H_3)_2$), 42.31 ($\underline{C}H_2Ph$), 51.37 ($\underline{C}HCH_3$), 61.33 ($\underline{C}H_2CH_3$), 70.83 (C4), 127.94 (Ar), 129.64 (Ar), 130.13 (Ar), 130.81 (Ar), 131.15 (Ar), 139.26 (Ar), 154.02 (C2), 168.35 (O=$\underline{C}$O), 208.40 ($\underline{C}$=S); LC/ESI-MS (m/z): positive mode 403 ($[M + H]^+$). Purity (HPLC-UV 282 nm): 98.4%.

General procedure D for regioselective cyclisation of 2-oxo-5-thioxoimidazolidines 20–22

A solution of the corresponding 2-oxo-5-thioxoimidazolidines **20**–**22** (1.00 equiv) in absolute ethanol (5–6 mL per 1.00 mmol starting material), hydrazine monohydrate (20.0 equiv), and freshly activated molecular sieve 3 Å (10.0 mg per 1.00 mmol starting material) were placed in a dry apparatus under an argon atmosphere. The reaction mixture was stirred under reflux for 5–10 h while complete conversion was detected by TLC control (eluent: dichloromethane/methanol, 95:5). After all of the starting material had disappeared, the solution was cooled to room temperature,

filtered and the solvent removed under reduced pressure. The remaining red oil was purified by column chromatography on silica gel and if necessary by recrystallization from dichloromethane/petroleum ether (1:9).

7-Benzyl-8,8-dimethyl-7,8-dihydroimidazo[5,1-*c*][1,2,4]triazine-3,6(2*H*,4*H*)-dione (23).

Following general procedure D 2-(3-benzyl-4,4-dimethyl-2-oxo-5-thioxoimidazolidin-1-yl)acetic acid ethyl ester (**20**; 123 mg, 0.38 mmol) was treated with hydrazine monohydrate (0.38 mL, 7.87 mmol). The product was obtained after column chromatography on silica gel (eluent: dichloromethane/methanol, 95:5, R_f = 0.49) and recrystallization from dichloromethane/petroleum ether as a white solid: Yield 92 mg (88%); mp 202.2–204.6 °C (dec.); ^{1}H NMR (500 MHz, DMSO-d_6, δ/ppm) 1.28 (s, 6H, C(C<u>H</u>$_3$)$_2$), 4.08 (s, 2H, C<u>H</u>$_2$, H-4), 4.45 (s, 2H, C<u>H</u>$_2$Ph), 7.22–7.27 (m, 1H, Ar), 7.30–7.37 (m, 4H, Ar), 10.54 (s, 1H, NH); ^{13}C NMR (125 MHz, DMSO-d_6, δ/ppm) 25.04 (2×CH_3, C(<u>C</u>H$_3$)$_2$), 42.02 (<u>C</u>H$_2$Ph), 42.04 (<u>C</u>H$_2$, C4)), 60.07 (C8), 127.20 (Ar), 127.55 (2×C, Ar), 128.42 (2×C, Ar), 138.83 (Ar), 148.20 (C9), 154.12 (C6), 159.29 (C3); LC/ESI-MS (*m*/*z*): negative mode 271 ([M − H]$^-$), positive mode 273 ([M + H]$^+$). Purity (HPLC-UV 276 nm): 99.1%; Anal. calcd. for $C_{14}H_{16}N_4O_2$: C, 61.75; H, 5.92; N, 20.58; found: C, 61.42; H, 6.33; N, 20.51.

7-Benzyl-4,8,8-trimethyl-7,8-dihydroimidazo[5,1-*c*][1,2,4]triazine-3,6(2*H*,4*H*)-dione (24).

Following general procedure D 2-(3-benzyl-4,4-dimethyl-2-oxo-5-thioxoimidazolidin-1-yl)propionic acid ethyl ester (**21**; 857 mg, 2.56 mmol) was treated with hydrazine monohydrate (2.55 mL, 52.5 mmol). The product was obtained after column chromatography on silica gel (eluent: dichloromethane/methanol, 95:5, R_f = 0.46) as

a white solid: Yield 645 mg (87%); mp 204.3–206.8 °C (dec.); NMR experiments: HMQC and HMBC; ^{1}H NMR (500 MHz, DMSO-d_6, δ/ppm) 1.26 (s, 3H, $C(C\underline{H}_3)_2$), 1.29 (s, 3H, $C(C\underline{H}_3)_2$), 1.46 (d, J = 6.62 Hz, 3H, $CHC\underline{H}_3$), 4.43 (q, J = 6.62 Hz, 1H, $C\underline{H}CH_3$), 4.45 (q, J = 16.08 Hz, 2H, $C\underline{H}_2Ph$), 7.22–7.26 (m, 1H, Ar), 7.30–7.35 (m, 4H, Ar), 10.54 (s, 1H, NH); ^{13}C NMR (125 MHz, DMSO-d_6, δ/ppm) 17.56 ($CH\underline{C}H_3$), 24.78 ($C(\underline{C}H_3)_2$), 25.20 ($C(\underline{C}H_3)_2$), 42.07 ($\underline{C}H_2Ph$), 49.22 ($\underline{C}HCH_3$), 59.59 (C8), 127.24 (Ar), 127.52 (2×C, Ar), 128.48 (2×C, Ar), 138.83 (Ar), 148.10 (C9), 154.03 (C6), 162.50 (C3); LC/ESI-MS (*m*/*z*): negative mode 285 ($[M - H]^-$), positive mode 287 ($[M + H]^+$). Purity (HPLC-UV 276 nm): 99.8%; Anal. calcd. for $C_{15}H_{18}N_4O_2$: C, 62.92; H, 6.34; N, 19.57; found: C, 63.38; H, 6.33; N, 19.51.

7-(3,4-Dichlorobenzyl)-4,8,8-trimethyl-7,8-dihydroimidazo[5,1-*c*][1,2,4]triazine-3,6(2*H*,4*H*)-dione (25).

Following general procedure D 2-[3-(3,4-dichlorobenzyl)-4,4-dimethyl-2-oxo-5-thioxoimidazolidin-1-yl]propionic acid ethyl ester (**22**; 661 mg, 1.64 mmol) was treated with hydrazine monohydrate (1.59 mL, 32.8 mmol). The product was obtained after column chromatography on silica gel (eluent: dichloromethane/methanol, 95:5, R_f = 0.49) and recrystallization from a mixture of dichloromethane and petroleum ether as a white solid: Yield 500 mg (86%); mp 194.8–195.8 °C (dec.); NMR experiments: HMQC and HMBC; ^{1}H NMR (500 MHz, DMSO-d_6, δ/ppm) 1.27 (s, 3H, $C(C\underline{H}_3)_2$), 1.33 (s, 3H, $C(C\underline{H}_3)_2$), 1.45 (d, J = 6.62 Hz, 3H, $CHC\underline{H}_3$), 4.43 (q, J = 6.62 Hz, 1H, $C\underline{H}CH_3$), 4.46 (q, J = 16.4 Hz, 2H, $C\underline{H}_2Ph$), 7.33 (dd, J = 1.57 / 8.19 Hz, 1H, Ar), 7.58 (d, J = 8.52 Hz, 1H, Ar), 7.62 (d, J = 1.26 Hz, 1H, Ar), 10.56 (s, 1H, NH); ^{13}C NMR (125 MHz, DMSO-d_6, δ/ppm) 17.57 ($CH\underline{C}H_3$), 24.65 ($C(\underline{C}H_3)_2$), 25.01 ($C(\underline{C}H_3)_2$), 40.92 ($\underline{C}H_2Ph$), 49.27 ($\underline{C}HCH_3$), 59.64 (C8), 127.90 (Ar), 129.57 (Ar), 129.80 (Ar), 130.66 (Ar), 131.05 (Ar), 140.21 (Ar), 147.88 (C9), 154.13 (C6), 162.48 (C3); LC/ESI-

MS (*m*/*z*): negative mode 353 ([M − H]$^-$), positive mode 355 ([M + H]$^+$). Purity (HPLC-UV 276 nm): 98.1%; Anal. calcd. for $C_{15}H_{18}N_4O_2$: C, 50.72; H, 4.54; N, 15.77; found: C, 50.38; H, 4.53; N, 15.65.

General procedure E for *N*-alkylation of imidazotriazines 24 and 25

A suspension of sodium hydride (60% in paraffin) was washed under an argon atmosphere with *n*-hexane (3 × 10 mL), and the solvent was removed under reduced pressure. The remaining material (1.26 equiv) of sodium hydride was suspended in anhydrous DMF (1–2 mL per 1.00 mmol starting material) under an argon atmosphere, stirred for 10 min at room temperature, and then treated with the corresponding imidazotriazine **24** (1.00 equiv) or **25**. The reaction mixture was stirred at room temperature for 15 min until hydrogen evolution was observed, and a solution of the appropriate alkylating reagent (1.26–2.10 equiv) in anhydrous DMF (0.25–0.30 mL per 1.00 mmol alkylation reagent) was added dropwise. The solution was stirred at room temperature (for alkylation with methyl iodide), or at 85 °C (for all other reactions), until complete conversion was detected by TLC (eluent: dichloromethane/methanol, 95:5). After all of the starting material had disappeared, the reaction mixture was diluted with water (10–30 mL), neutralized with 2N HCl and extracted with dichloromethane (3 × 50 mL). The combined organic layers were dried over sodium sulfate, filtered and evaporated under reduced pressure. The crude products were purified by column chromatography on silica gel followed by recrystallization from dichloromethane/petroleum ether (1:9) or by RP-HPLC.

7-Benzyl-2,4,8,8-tetramethyl-7,8-dihydroimidazo[5,1-*c*][1,2,4]triazine-3,6(2*H*,4*H*)-dione (26).

Following general procedure E, 7-benzyl-4,8,8-trimethyl-7,8-dihydroimidazo[5,1-*c*]-[1,2,4]triazine-3,6(2*H*,4*H*)-dione (**24**; 200 mg, 0.70 mmol) was treated with methyl iodide (297 mg, 2.10 mmol). The reaction mixture was stirred at room temperature for 72 h. Pure product was obtained after column chromatography on silica gel (eluent: dichloromethane/methanol, 95:5, R_f = 0.58) followed by RP-HPLC (eluent: methanol/water, 70:30) and recrystallization from dichloromethane/petroleum ether (9:1), as a white solid: yield 120 mg (57%); mp 70.4–71.2 °C (dec.); ^{1}H NMR (500 MHz, DMSO-d_6, δ/ppm) 1.28 (s, 3H, C(C$\underline{H}_3$)$_2$), 1.31 (s, 3H, C(C$\underline{H}_3$)$_2$), 1.46 (d, *J* = 6.62 Hz, 3H, CHC$\underline{H}_3$), 3.17 (s, 3H, NC$\underline{H}_3$), 4.46 (q, *J* = 16.39 Hz, 2H, C$\underline{H}_2$Ph), 4.48 (q, *J* = 7.25 Hz, 1H, C$\underline{H}$CH$_3$), 7.22–7.27 (m, 1H, Ar), 7.30–7.35 (m, 4H, Ar); ^{13}C NMR (125 MHz, DMSO-d_6, δ/ppm) 17.77 (CH$\underline{C}$H$_3$), 24.78 (C($\underline{C}$H$_3$)$_2$), 25.16 (C($\underline{C}$H$_3$)$_2$), 36.51 (N$\underline{C}$H$_3$), 42.06 ($\underline{C}$H$_2$Ph), 49.32 ($\underline{C}$HCH$_3$), 59.82 (C8), 127.26 (Ar), 127.53 (2×C, Ar), 128.48 (2×C, Ar), 138.71 (Ar), 148.33 (C9), 153.78 (C6), 160.54 (C3); LC/ESI-MS (*m*/*z*): positive mode 301 ([M + H]$^+$). Purity (HPLC-UV 276 nm): 98.8%; Anal. calcd. for $C_{16}H_{20}N_4O_2$: C, 63.98; H, 6.71; N, 18.65; found: C, 63.54; H, 6.67; N, 18.51.

7-(3,4-Dichlorobenzyl)-2,4,8,8-tetramethyl-7,8-dihydroimidazo[5,1-*c*][1,2,4]-triazine-3,6(2*H*,4*H*)-dione (27).

Following general procedure E, 7-(3,4-dichlorobenzyl)-4,8,8-trimethyl-7,8-dihydro-imidazo-[5,1-*c*][1,2,4]triazine-3,6(2*H*,4*H*)-dione (**25**; 150 mg, 0.42 mmol) was treated with methyl methanesulfonate (59.0 mg, 0.54 mmol). The reaction mixture was stirred at room temperature for 24 h. The product was obtained after purification by column chromatography on silica gel (eluent: dichloromethane/methanol, 95:5, R_f = 0.60)

following by RP-HPLC (eluent: methanol/water, 70:30), as a white solid: Yield 140 mg (88%); mp 114.8 – 115.5 °C (dec.); ^{1}H NMR (500 MHz, DMSO-d_6, δ/ppm) 1.29 (s, 3H, $C(C\underline{H}_3)_2$), 1.35 (s, 3H, $C(C\underline{H}_3)_2$), 1.46 (d, J = 6.93 Hz, 3H, $CHC\underline{H}_3$), 3.17 (s, 3H, $NC\underline{H}_3$), 4.46 (q, J = 16.4 Hz, 2H, $C\underline{H}_2Ph$), 4.49 (q, J = 6.62 Hz, 1H, $C\underline{H}CH_3$), 7.33 (dd, J = 1.89 / 8.19 Hz, 1H, Ar), 7.58 (d, J = 8.19 Hz, 1H, Ar), 7.62 (d, J = 1.90 Hz, 1H, Ar); ^{13}C NMR (125 MHz, DMSO-d_6, δ/ppm) 17.80 ($CH\underline{C}H_3$), 24.63 ($C(\underline{C}H_3)_2$), 24.97 ($C(\underline{C}H_3)_2$), 36.52 ($N\underline{C}H_3$), 40.90 ($\underline{C}H_2Ph$), 49.37 ($\underline{C}HCH_3$), 59.86 (C8), 127.90 (Ar), 129.57 (Ar), 129.82 (Ar), 130.67 (Ar), 131.06 (Ar), 140.10 (Ar), 148.12 (C9), 153.88 (C6), 160.54 (C3); LC/ESI-MS (*m*/*z*): positive mode 368 ($[M + H]^+$). Purity (HPLC-UV 276 nm): 99.9%; Anal. calcd. for $C_{16}H_{18}Cl_2N_4O_2$: C, 52.04; H, 4.91; N, 15.77; found: C, 51.61; H, 4.94; N, 15.34.

7-Benzyl-4,8,8-trimethyl-2-(prop-2-ynyl)-7,8-dihydroimidazo[5,1-*c*][1,2,4]triazine-3,6(2*H*,4*H*)-dione (28).

Following general procedure E, 7-benzyl-4,8,8-trimethyl-7,8-dihydroimidazo[5,1-*c*][1,2,4]triazine-3,6(2*H*,4*H*)-dione (**24**; 300 mg, 1.05 mmol) was treated with propargyl bromide (80% solution in toluene; 188 mg, 1.32 mmol). The reaction mixture was stirred at 85 °C for 48 h. The product was obtained after purification by column chromatography on silica gel (eluent: dichloromethane/methanol, 95:5, R_f = 0.83) and subsequent recrystallization from dichloromethane/petroleum ether (9:1), as a white solid: yield 312 mg (92%); mp 127.2–128.1 °C (dec.); ^{1}H NMR (500 MHz, DMSO-d_6, δ/ppm) 1.32 (s, 3H, $C(C\underline{H}_3)_2$), 1.35 (s, 3H, $C(C\underline{H}_3)_2$), 1.60 (d, J = 6.62 Hz, 3H, $CHC\underline{H}_3$), 2.19 (t, J = 2.20 Hz, 1H, $CH_2CC\underline{H}$), 4.43 (dt, J = 2.52 / 17.03 Hz, 2H, $C\underline{H}_2CCH$), 4.46 (q, J = 15.44 Hz, 2H, $C\underline{H}_2Ph$), 4.64 (q, J = 6.62 Hz, 1H, $C\underline{H}CH_3$), 7.23–7.33 (m, 5H, Ar); ^{13}C NMR (125 MHz, DMSO-d_6, δ/ppm) 17.99 ($CH\underline{C}H_3$), 25.31 ($C(\underline{C}H_3)_2$), 25.54 ($C(\underline{C}H_3)_2$), 38.42 ($\underline{C}H_2CCH$), 43.05 ($\underline{C}H_2Ph$), 49.91 ($\underline{C}HCH_3$), 60.27

(C8), 71.51 ($CH_2C\underline{C}H$), 78.17 ($CH_2\underline{C}CH$), 127.75 (3×C, Ar), 128.69 (2×C, Ar), 137.84 (Ar), 148.89 (C9), 153.85 (C6), 160.59 (C3); LC/ESI-MS (*m*/*z*): positive mode 325 ($[M + H]^+$). Purity (HPLC-UV 276 nm): 98.7%; Anal. calcd. for $C_{18}H_{20}N_4O_2$: C, 66.65; H, 6.21; N, 17.27; found: C, 66.22; H, 6.28; N, 16.81.

7-(3,4-Dichlorobenzyl)-4,8,8-trimethyl-2-(prop-2-ynyl)-7,8-dihydroimidazo[5,1-*c*]-[1,2,4]triazine-3,6(2*H*,4*H*)-dione (29).

Following general procedure E, 7-(3,4-dichlorobenzyl)-4,8,8-trimethyl-7,8-dihydro-imidazo[5,1-*c*][1,2,4]triazine-3,6(2*H*,4*H*)-dione (**25**; 150 mg, 0.42 mmol) was treated with propargyl bromide (80% solution in toluene; 79.0 mg, 0.55 mmol). The reaction mixture was stirred at 85 °C for 48 h. The product was obtained after purification by column chromatography on silica gel (eluent: dichloromethane/methanol, 95:5, R_f = 0.78) followed by RP-HPLC (eluent: methanol/water, 70:30) as a white solid: Yield 53 mg (68%); mp 99.5–101.7 °C (dec.); ^1H NMR (500 MHz, DMSO-d_6, δ/ppm) 1.30 (s, 3H, $C(C\underline{H}_3)_2$), 1.37 (s, 3H, $C(C\underline{H}_3)_2$), 1.48 (d, J = 6.93 Hz, 3H, $CHC\underline{H}_3$), 3.16 (t, J = 2.53 Hz, $CH_2CC\underline{H}$), 4.38 (d, J = 2.52 Hz, 2H $C\underline{H}_2CCH$), 4.47 (q, J = 16.71 Hz, 2H, $C\underline{H}_2Ph$), 4.56 (q, J = 6.62 Hz, 1H, $C\underline{H}CH_3$), 7.35 (dd, J = 1.89 / 8.19 Hz, 1H, Ar), 7.58 (d, J = 8.20 Hz, 1H, Ar), 7.63 (d, J = 1.89 Hz, 1H, Ar); ^{13}C NMR (125 MHz, DMSO-d_6, δ/ppm) 17.69 ($CH\underline{C}H_3$), 24.66 ($C(\underline{C}H_3)_2$), 24.95 ($C(\underline{C}H_3)_2$), 38.15 ($\underline{C}H_2CCH$), 40.92 ($\underline{C}H_2Ph$), 49.47 ($\underline{C}HCH_3$), 59.94 (C8), 74.23 ($CH_2C\underline{C}H$), 79.18 ($CH_2\underline{C}CH$), 127.87 (Ar), 129.54 (Ar), 129.80 (Ar), 130.64 (Ar), 131.06 (Ar), 140.03 (Ar), 148.82 (C9), 153.72 (C6), 160.38 (C3); LC/ESI-MS (*m*/*z*): positive mode 393 ($[M + H]^+$). Purity (HPLC-UV 276 nm): 98.6%; Anal. calcd. for $C_{18}H_{18}Cl_2N_4O_2$: C, 54.97; H, 4.81; N 14.25; found: C, 54.89; H, 5.26; N, 14.03.

NMR and LC/ESI-MS spectra of compounds 24 and 25

7-Benzyl-4,8,8-trimethyl-7,8-dihydroimidazo[5,1-*c*][1,2,4]triazine-3,6(2*H*,4*H*)-dione (**24**)

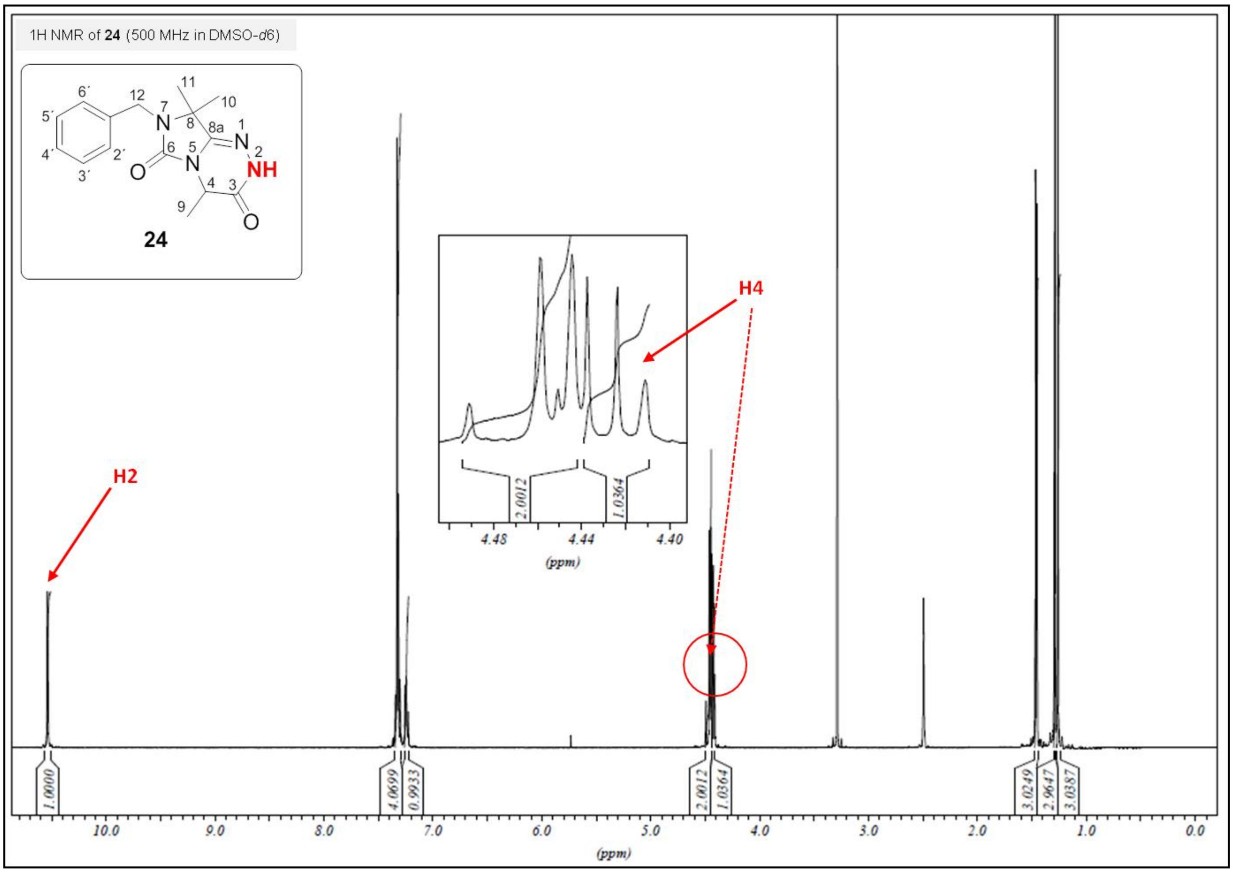

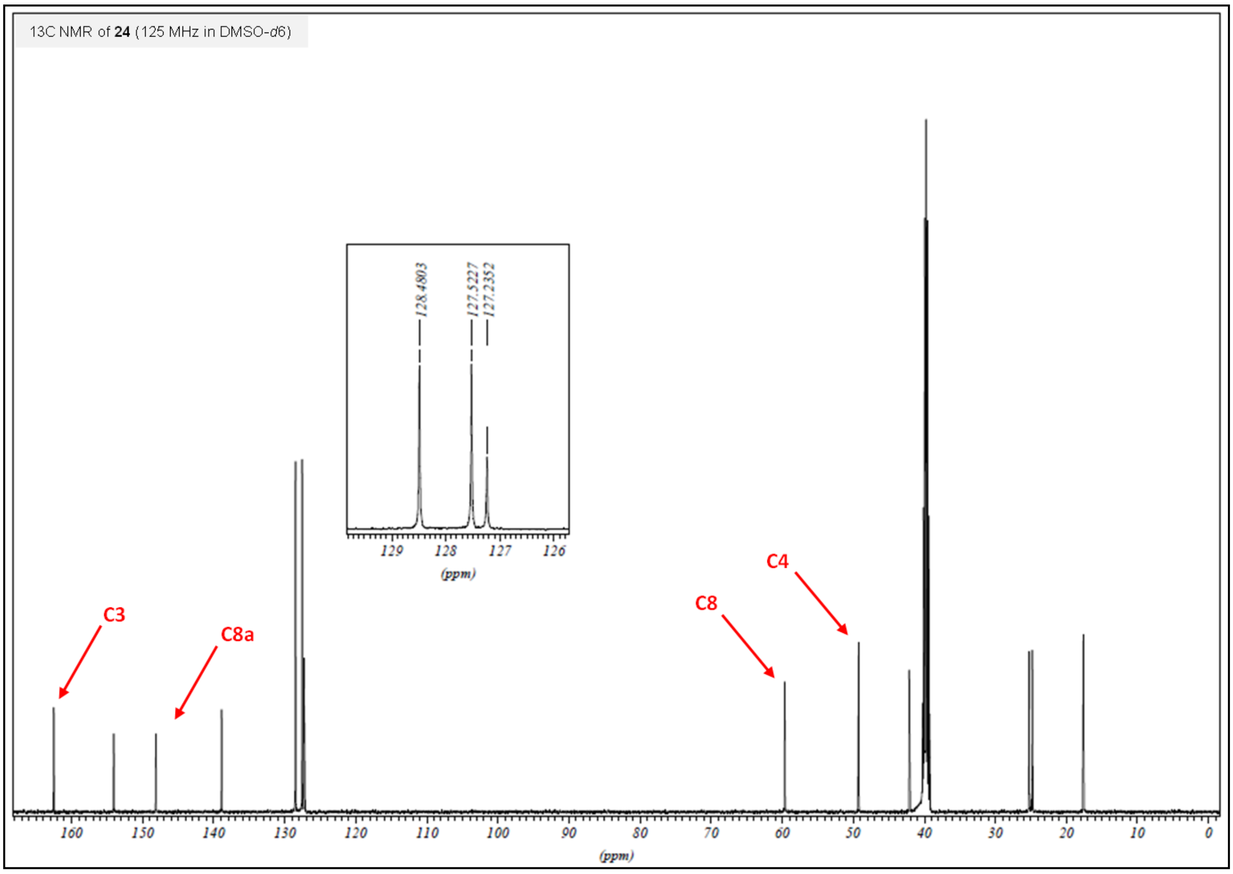

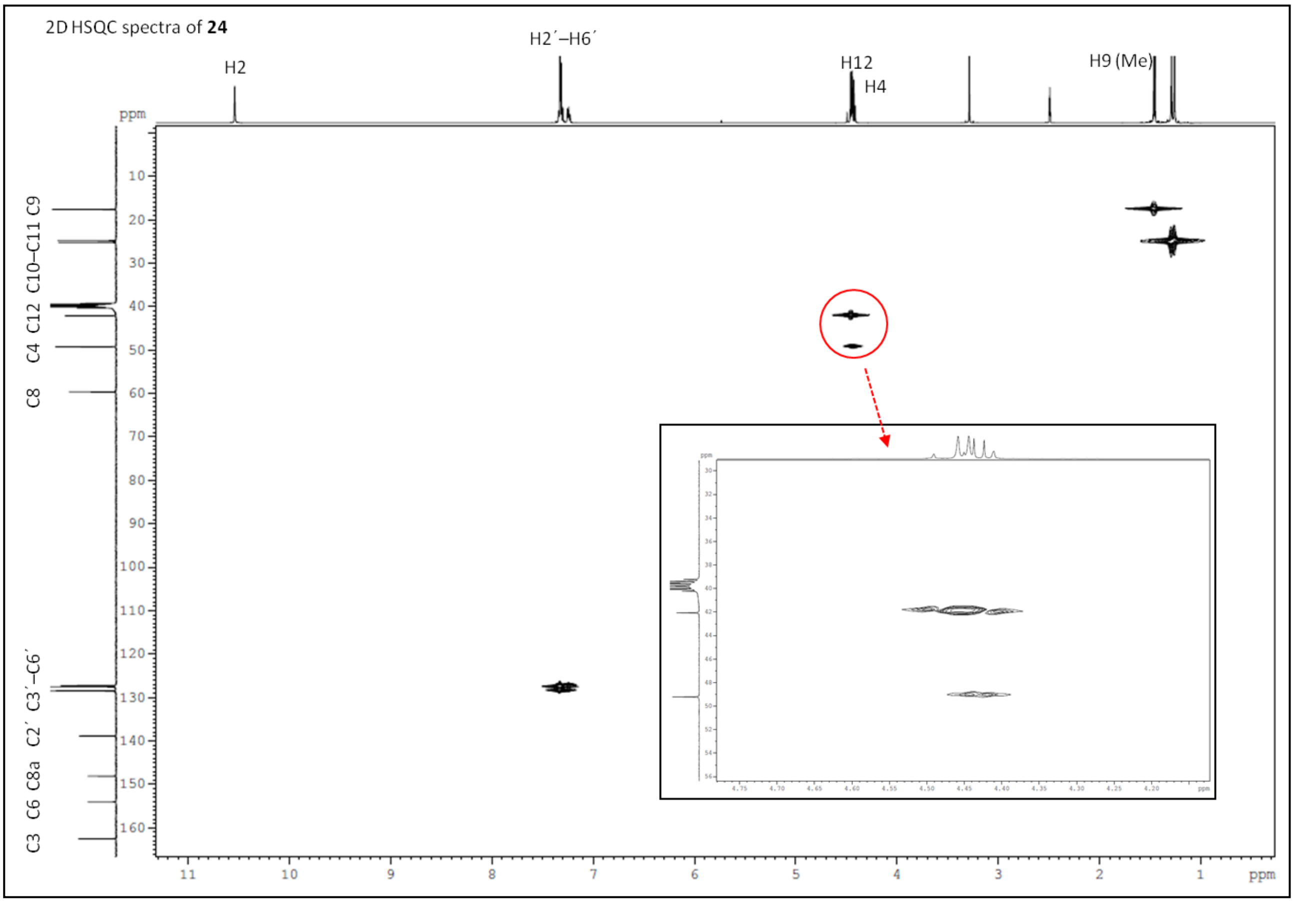
2D HSQC spectra of **24**
H2
H2´–H6´
H12
H4
H9 (Me)
C9
C10–C11
C12
C4
C8
C3´–C6´
C2´
C8a
C6
C3
ppm

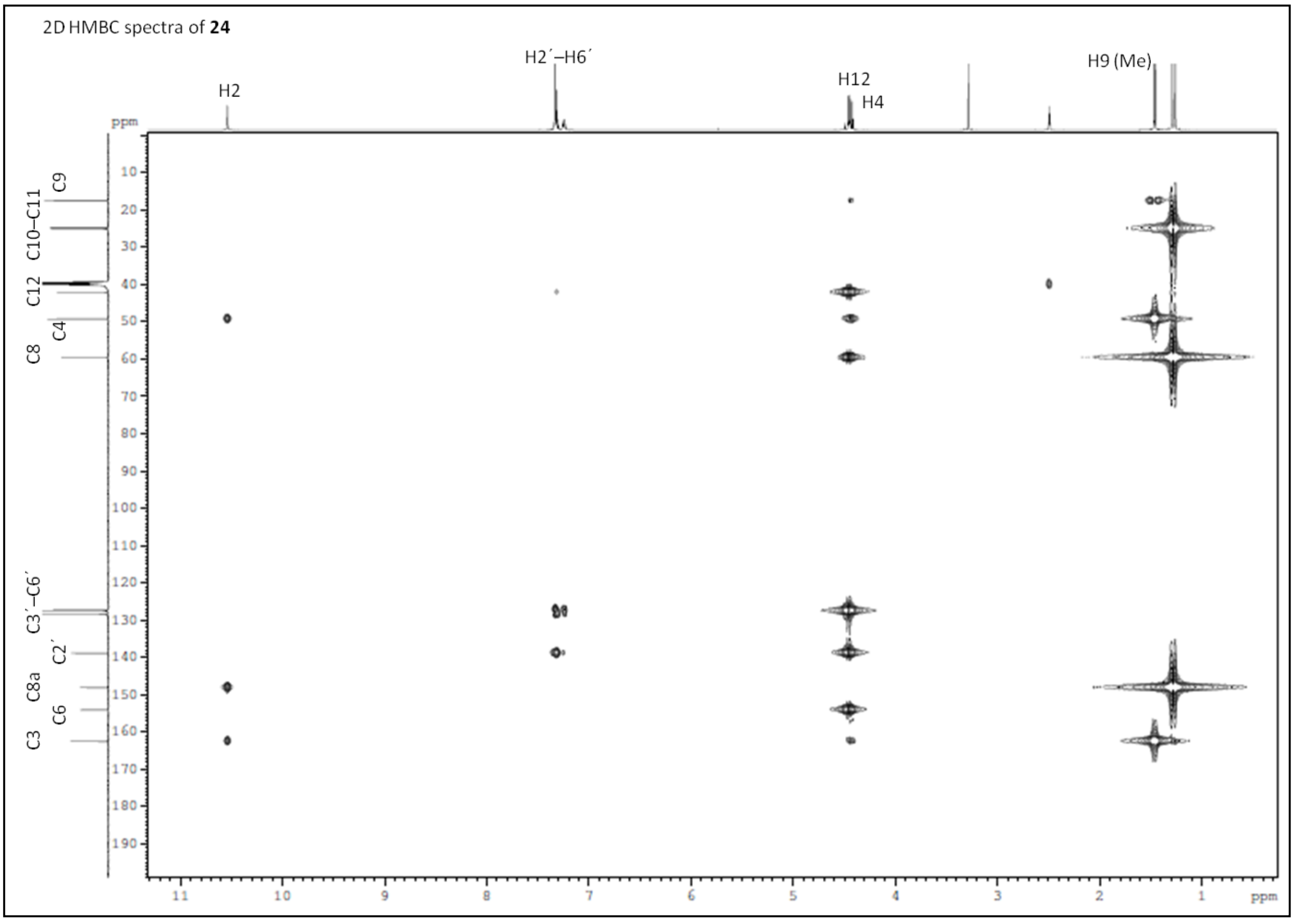
2D HMBC spectra of **24**
H2
H2´–H6´
H12
H4
H9 (Me)
C9
C10–C11
C12
C4
C8
C3´–C6´
C2´
C8a
C6
C3
ppm

Sample Name: NT01304
Acq. Date: Thursday, January 12, 2012
Acq. File: 2012-01-12.wiff
Polarity/Scan Type: Positive Q1 MS, ...
Scan Mass(es): Start: 150.0, Stop: 800.0, Step: 0.1

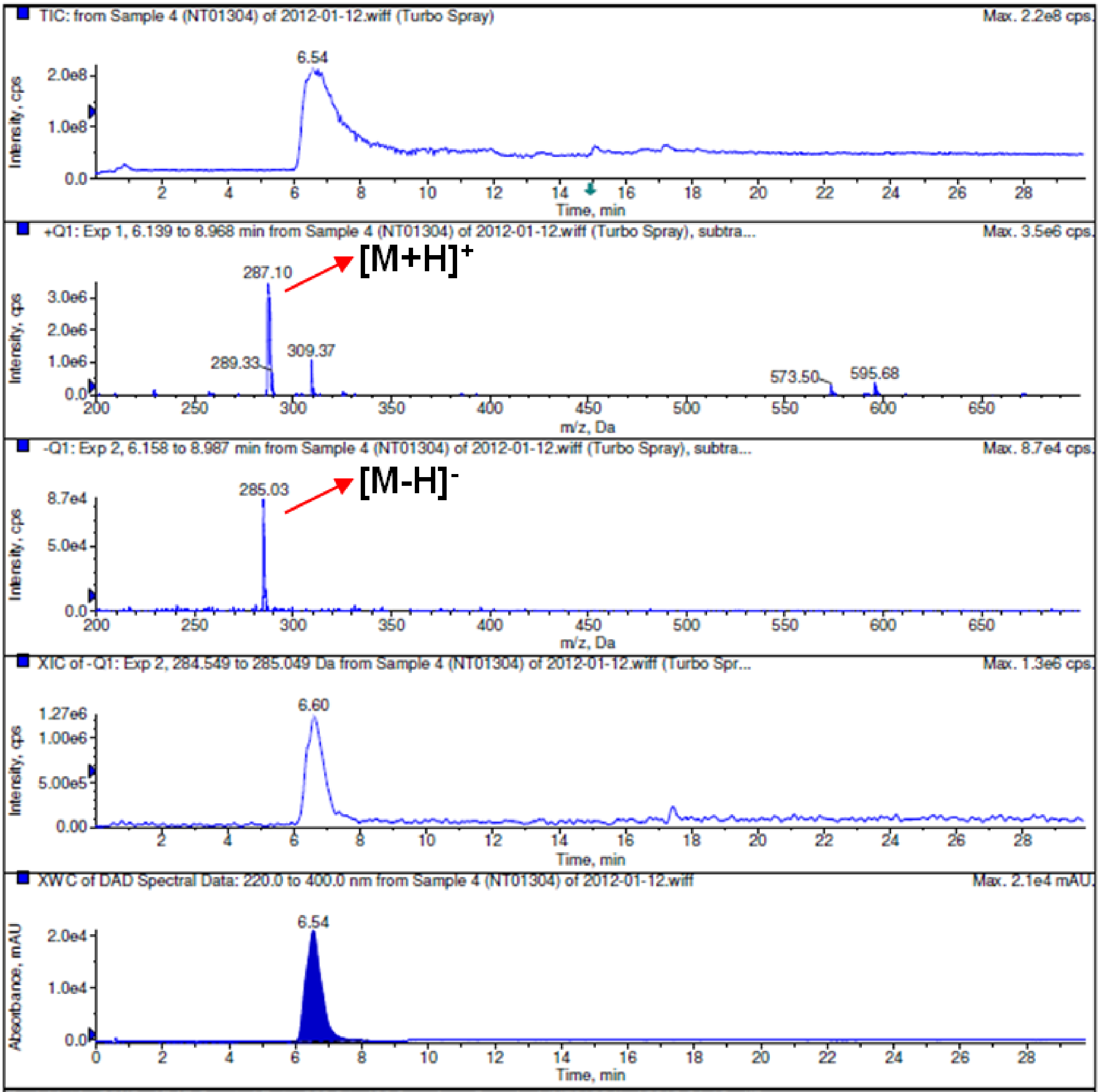

Peak List for "XWC of DAD Spectral Data: 220.0 to 400.0 nm from Sample 4 (NT01304) of 2012-01-12.wiff"

	Time (min)	Area (mAU x min)	% Area	Height (mAU)	% Height	Width (min)	Baseline Type
1	6.5387	6.8002e5	100.0000	2.1309e4	100.0000	2.2933	Base to Base

LC Pump Device: Agilent 1100 binary pump

7-(3,4-Dichlorobenzyl)-4,8,8-trimethyl-7,8-dihydroimidazo[5,1-*c*][1,2,4]triazine-3,6(2*H*,4*H*)-dione (**25**)

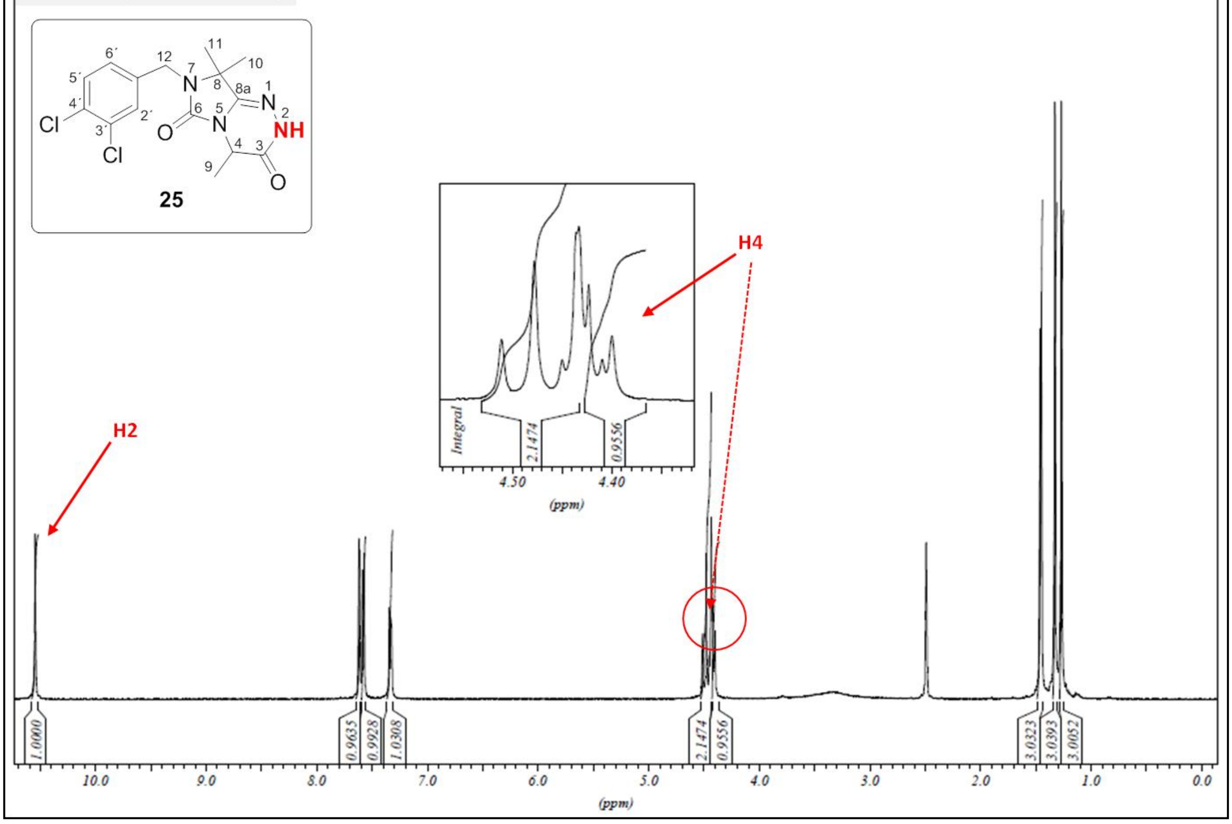

1H NMR of **25** (500 MHz in DMSO-*d*6)

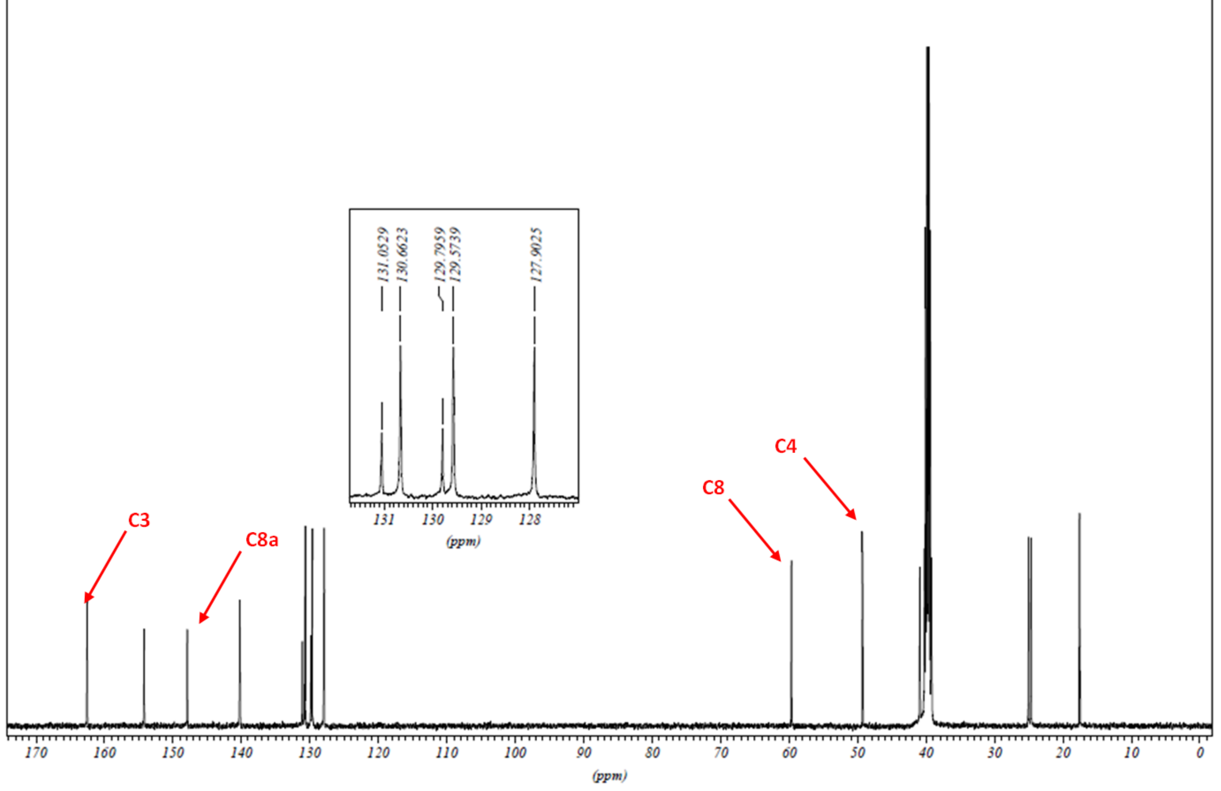

13C NMR of **25** (125 MHz in DMSO-*d*6)

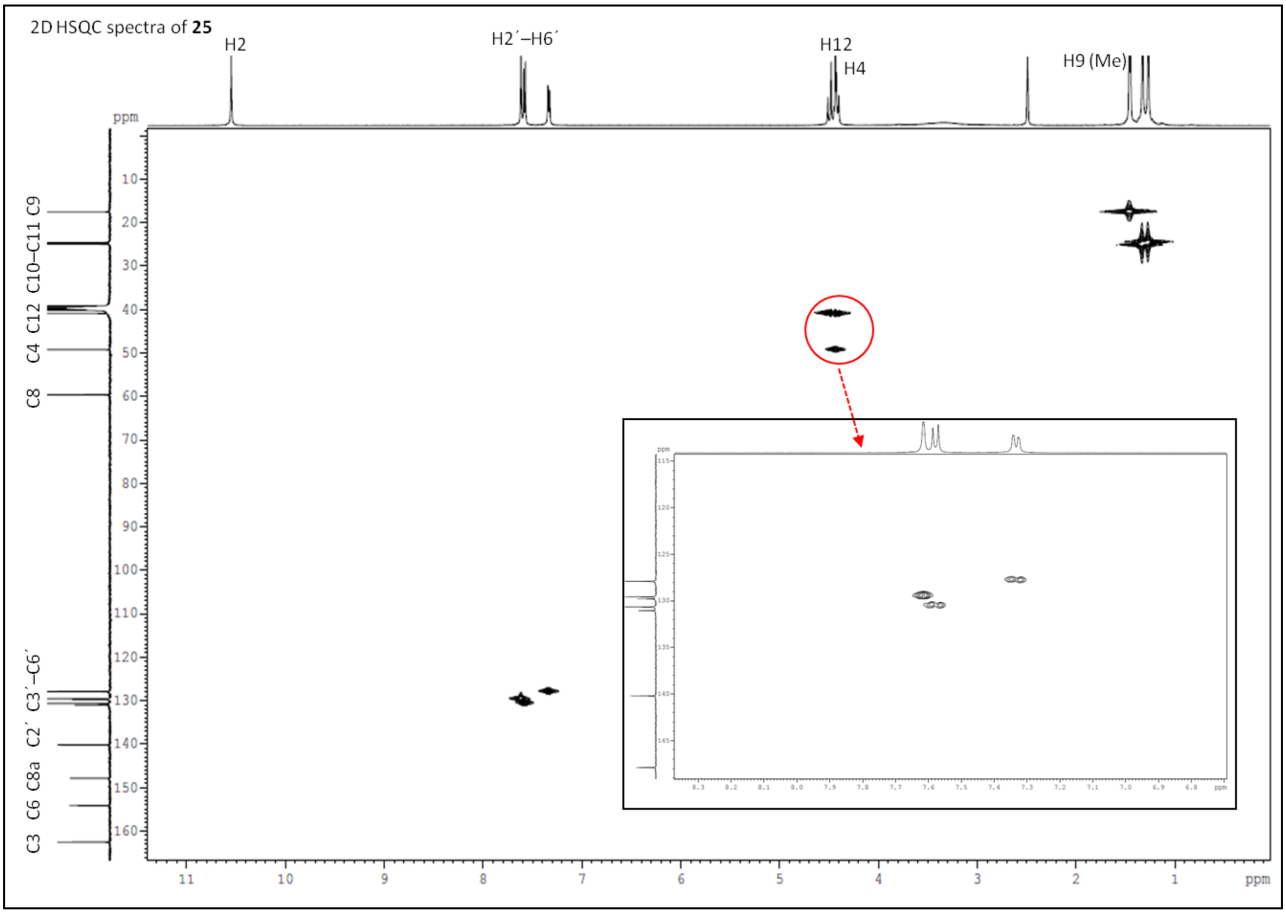
2D HSQC spectra of **25**
H2
H2´–H6´
H12
H4
H9 (Me)
C9
C10–C11
C12
C4
C8
C3´–C6´
C2´
C8a
C6
C3
ppm

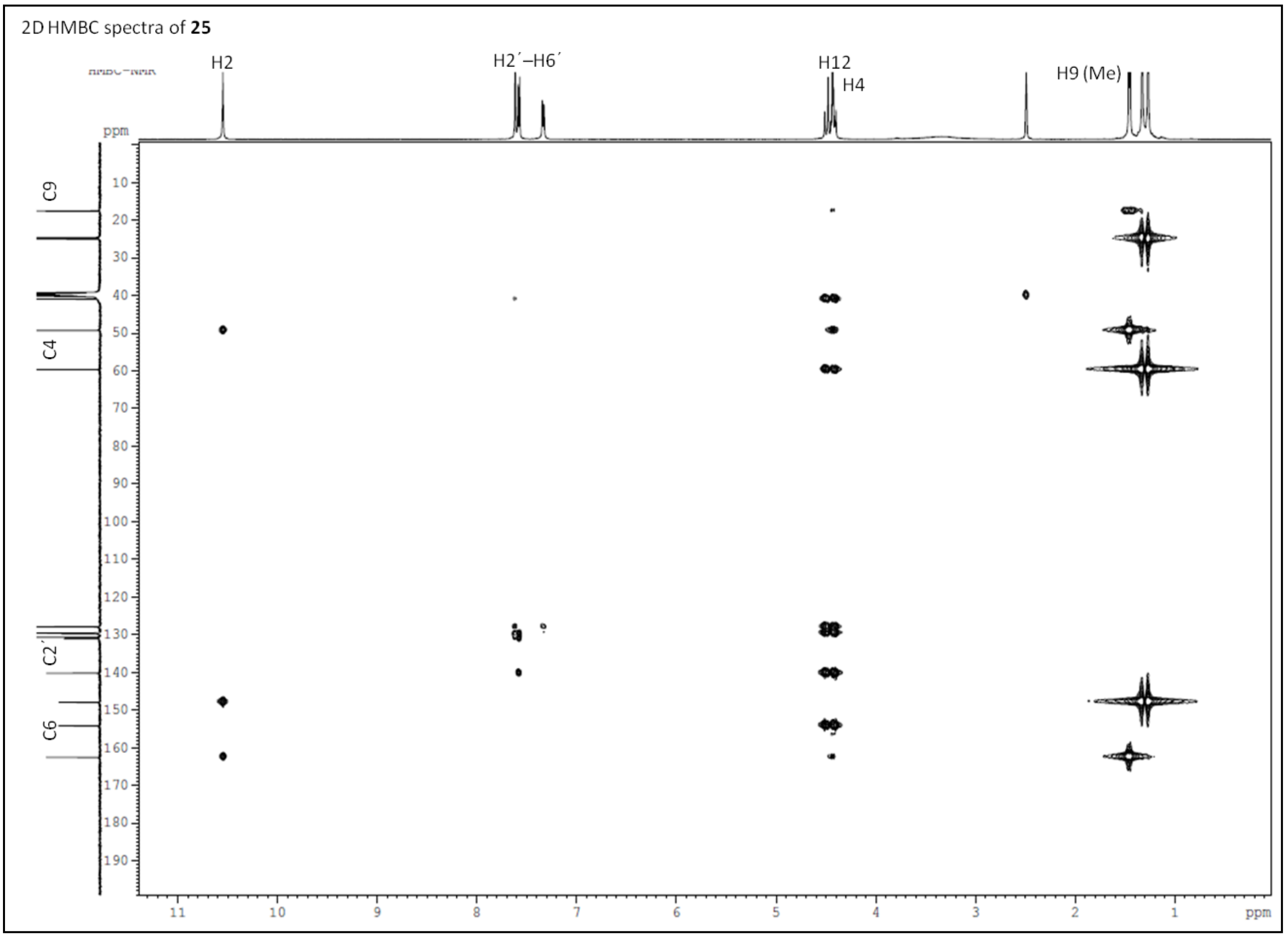
2D HMBC spectra of **25**
H2
H2´–H6´
H12
H4
H9 (Me)
C9
C4
C2´
C6
ppm

Sample Name: NT01803 Polarity/Scan Type: Positive Q1 MS, ...
Acq. Date: Wednesday, February 22, 2012 Scan Mass(es): Start: 150.0, Stop: 800.0, Step: 0.1
Acq. File: 2012-02-21.wiff

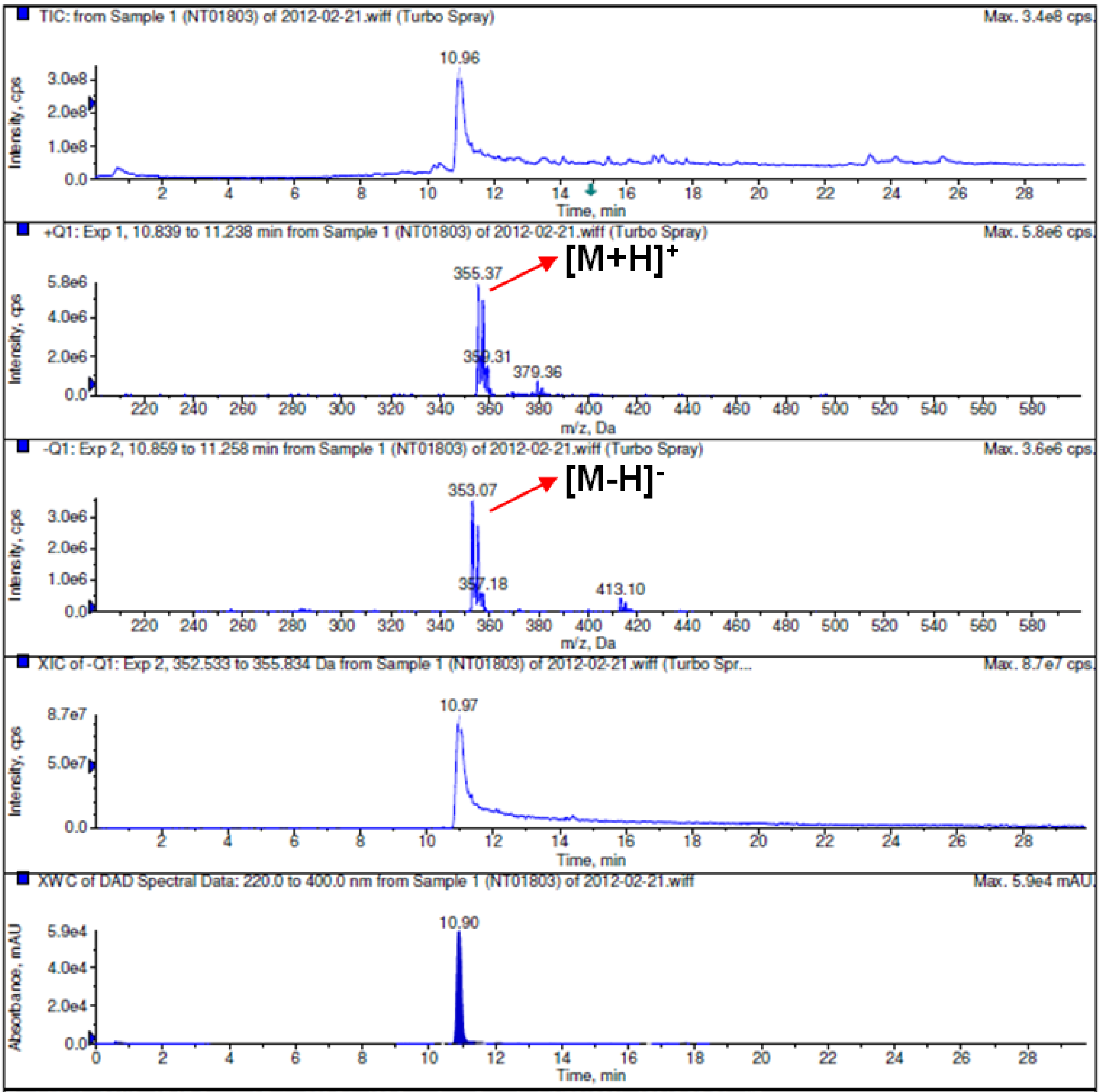

Peak List for "XWC of DAD Spectral Data: 220.0 to 400.0 nm from Sample 1 (NT01803) of 2012-02-21.wiff"

	Time (min)	Area (mAU x min)	%Area	Height (mAU)	%Height	Width (min)	Baseline Type
1	0.7066	739.8608	0.1373	112.0790	0.1860	0.2600	Base to Base
2	10.3257	1521.3040	0.2823	174.8875	0.2903	0.3333	Base to Base
3	10.9050	5.3163e5	98.6486	5.9316e4	98.4506	0.7400	Base to Base
4	11.5398	1563.3786	0.2901	233.5076	0.3876	0.2600	Base to Base
5	12.0783	703.2710	0.1305	106.7886	0.1772	0.2533	Base to Base
6	16.5205	684.7139	0.1271	100.5567	0.1669	0.2867	Base to Base
7	17.7260	2070.3039	0.3842	205.6846	0.3414	0.4800	Base to Base

LC Pump Device: Agilent 1100 binary pump
